# Geminal Brønsted Acid Ionic Liquids as Catalysts for the Mannich Reaction in Water

**DOI:** 10.3390/ijms15058656

**Published:** 2014-05-15

**Authors:** Leqin He, Shenjun Qin, Tao Chang, Yuzhuang Sun, Jiquan Zhao

**Affiliations:** 1 School of Chemical Engineering, Hebei University of Technology, Tianjin 300130, China; E-Mail: heleqin@163.com; 2 Key Laboratory for Resource Exploration Research of Hebei Province, Hebei University of Engineering, Handan 056038, China; E-Mails: qinsj528@hebeu.edu.cn (S.Q.); sun_yz@hotmail.com (Y.S.)

**Keywords:** Mannich reaction, geminal ionic liquid, water, catalyst, emulsion

## Abstract

Quaternary ammonium geminal Brønsted acid ionic liquids (GBAILs) based on zwitterionic 1,2-bis[*N*-methyl-*N*-(3-sulfopropyl)-alkylammonium]ethane (where the carbon number of the alkyl chain is 4, 8, 10, 12, 14, 16, or 18) and *p*-toluenesulfonic acid monohydrate were synthesized. The catalytic ionic liquids were applied in three-component Mannich reactions with an aldehyde, ketone, and amine at 25 °C in water. The effects of the type and amount of catalyst and reaction time as well as the scope of the reaction were investigated. Results showed that GBAIL-C_14_ has excellent catalytic activity and fair reusability. The catalytic procedure was simple, and the catalyst could be recycled seven times via a simple separation process without noticeable decreases in catalytic activity.

## 1. Introduction

The Mannich reaction is one of the most important carbon–carbon bond-forming reactions in organic synthesis because of its atom economy and potential application in the synthesis of biologically active molecules [1–3]. In this reaction, an amine, two carbonyl compounds, and acid (or base) catalysts are used to produce β-amino carbonyl compounds, which constitute various pharmaceuticals, natural products, and versatile synthetic intermediates [4,5]. Conventional catalysts for the classic Mannich reaction involve inorganic and organic acids like HCl [6], proline [7], *p*-dodecylbenzenesulfonic acid [8], and several Lewis acids [9]. Reactions using these catalysts, however, often suffer from drawbacks including long reaction times, harsh reaction conditions, and difficult product separation. Utilization of solid catalysts may solve problems associated with current catalysts [10–12]. However, rapid deactivation and decreases in active sites per area limit the applications of solid catalysts to some degree. Therefore, new green catalysts are of great interest in this field.

Considering the focus on green synthesis in recent years, ionic liquid (ILs), including functionalized ones, have attracted considerable research attention [13–17]. ILs have been referred to as “designer solvents” because their physical and chemical properties can be adjusted by varying the cation and anion. These useful materials not only dissolve many organic and inorganic substances but are also readily recycled. Moreover, IL properties can be tuned to implement specific chemical tasks. Scientists have performed the Mannich reaction using various types of ILs. In some instances, catalysts and substrates may be dissolved into nearly neutral ILs, such as [BMIM][PF_6_] [18], [emim][OTf] [19], and [Hmim][PF_6_] [20] to promote the one-pot Mannich reaction. However, in most cases, acidic ILs are used both as the reaction medium and catalyst. Li *et al.* [21] reported a Mannich reaction catalyzed by a carboxyl-functionalized acidic IL [CMMIM][BF_4_] in aqueous media. The use of the IL 2-pyrrolidinecarboxylic acid as a highly efficient organocatalyst for the asymmetric one-pot Mannich reaction has also been reported by Wang *et al.* [22]. Other researchers have reported that the Mannich reaction could be conducted using Brønsted acid ILs as catalysts and solvents [23–25]. Although extensive work has been conducted in this area, the disadvantages of the aforementioned catalytic systems, including the large amount of catalyst required and the necessity of an organic co-solvent, are evident. Moreover, the systems typically contain halogen and metal atoms, which, in some ways, limits their “greenness”. Thus, synthesizing halogen-free and water-soluble ILs that can be used in a precise manner is necessary.

Organic reactions in water without the use of harmful organic solvents have recently become the focus of considerable research because water is a cheap, safe, and environmentally benign solvent. Some catalytic systems have been successfully applied in three-component Mannich-type reactions with aldehydes, amines, and ketones in water [26–29]. However, a major disadvantage in the use of an aqueous solvent is that most organic compounds are insoluble in water. To circumvent this disadvantage, surfactant-type catalysts [30,31], which solubilize organic materials or form micellar dispersions, have been utilized.

ILs with the molecular structure of gemini surfactants (geminal ILs), which are well known to show high surface activities, have gained significant research interest [32–34]. A number of reports have described the aggregation of these surface-active ILs in water. Therefore, geminal ILs may have potential applications in many areas.

**Scheme 1.**
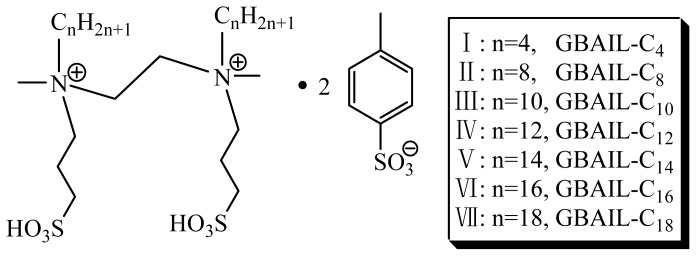
Structures of the geminal Brønsted acid ionic liquids (GBAILs).

In the present work, novel quaternary ammonium geminal Brønsted acid ionic liquids (GBAILs) were synthesized (see Scheme 1) and applied to catalyze three-component Mannich reactions in water. The ILs were characterized using IR, ^1^H-NMR, and ^13^C-NMR. Factors affecting the reaction, such as the type and amount of catalyst used and the reaction time, were investigated, and microscopic observations of colloidal particles created using GBAIL and organic substrates are presented an increase in the hydrocarbon chain length of the catalyst resulted in increases in product yield. To the best of our knowledge, Mannich reactions catalyzed by geminal ILs have yet to be reported.

## 2. Results and Discussion

### 2.1. Catalyst Screening for the Mannich Reaction

Mannich reactions of benzaldehyde, aniline, and cyclohexanone were chosen as model reactions to test the catalytic activities of GBAILs. The results are summarized in Table 1.

All reactions produce moderate to high yields, which suggests that the GBAILs behave as Brønsted acids, surfactants, and effective catalysts for Mannich-type reactions in water. An increase in the length of the hydrocarbon chain of the catalyst resulted in an increased product yield (entries 1–5). The product yield was stable when the number of carbon atoms in the hydrophobic chain exceeded 14 (entries 6 and 7). These results show that long alkyl chains are crucial for efficient catalysis.

**Figure 1.**
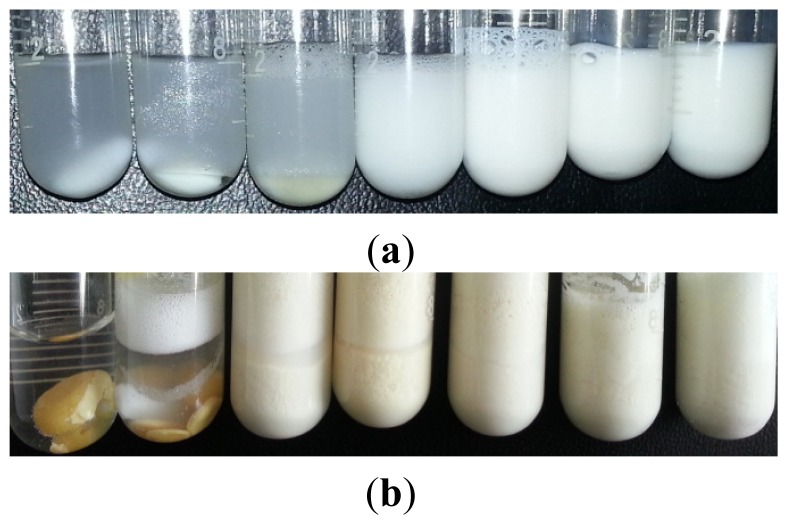
Different stages of the Mannich reaction catalyzed by GBAILs. (**a**) During the reaction; (**b**) at the end of reaction. The order of ionic liquids (ILs) from left to right is GBAIL-C_4_, GBAIL-C_8_, GBAIL-C_10_, GBAIL-C_12_, GBAIL-C_14_, GBAIL-C_16_, and GBAIL-C_18_.

As shown in Figure 1a, the seven reaction tubes were stirred for 30 min at 25 °C. After standing for 1 min, turbid emulsions induced by GBAILs with short alkyl chains (less than C_10_) formed two immiscible layers. By contrast, reaction mixtures with GBAILs with modest alkyl chain lengths (C_12_ or C_14_) presented white turbid reaction mixtures after standing for 1 h. No phase separation was observed in the turbid emulsions induced by GBAIL-C_16_ or GBAIL-C_18_ after standing for 10 h. After completion (Figure 1b), waxy solids were observed in reactions catalyzed by GBAIL-C_4_, GBAIL-C_8_, and GBAIL-C_10_, which may be due to low reaction rates in these systems. When the number of carbons in the hydrophobic chain exceeded 12, the particles became smaller with increasing hydrocarbon chain length. This result shows that long alkyl chains are necessary to form the colloidal dispersions assumed to promote efficient catalysis. Thus, considering the reaction rate and catalyst costs, GBAIL-C_14_ was selected as the most efficient catalyst for the Mannich reaction.

### 2.2. Reaction Conditions of the Mannich Reaction

Reactions of benzaldehyde, cyclohexanone, and aniline were used as model reactions to screen the effect of the amount of catalytic GBAIL-C_14_ (Table 2) on product yield. When the catalyst loading was increased from 1% to 5%, the product yield increased significantly from 43% to 88% (entries 1–5). The optimal amount of IL catalyst was 0.050 mmol (5 mol % based on the substrate), and further increases in catalyst amount did not substantially improve yields (entries 6 and 7). Therefore, we conclude that a sufficient number of active sites is available for the reaction to occur when the catalyst loading is 0.050 mmol. The effect of reaction time on the yield was also investigated. An increase in conversion was observed as the reaction time increased (entries 8–11). Further increases in reaction time only increased the product yield slightly (entries 12–14). This finding can be explained by the fact that the Mannich reaction reaches equilibrium in approximately 5 h. As such, 5 h was considered the ideal reaction time.

**Figure 2.**
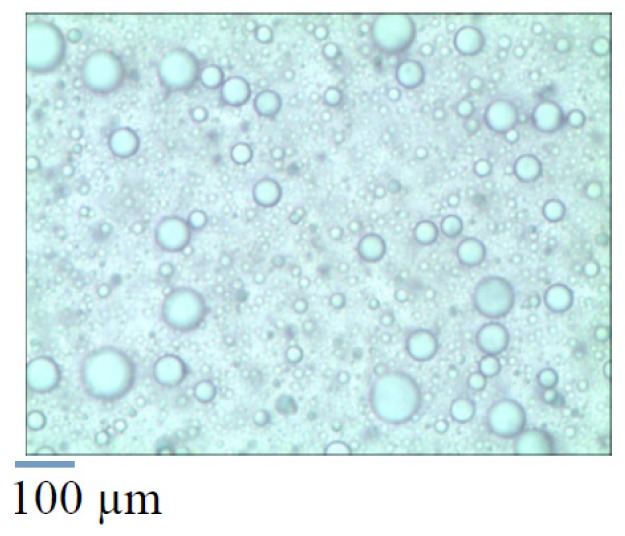
Optical micrograph of the reaction mixture.

The reaction mixtures became turbid with increasing reaction time, which signifies the formation of colloidal dispersions. We observed the GBAIL-C_14_-induced colloid via optical microscopy of the colloidal dispersions formed from GBAIL-C_14_ (5 mol %), benzaldehyde (1.0 mol), and aniline (1.0 mol) in water (1.5 mL) and found that spherical particles were formed (Figure 2). Most of the substrate and catalyst are presumably concentrated in the spherical particles, which function as hydrophobic reaction sites and enable rapid reaction in water.

### 2.3. Reusability of GBAIL-C_14_ in the Mannich Reaction

**Figure 3.**
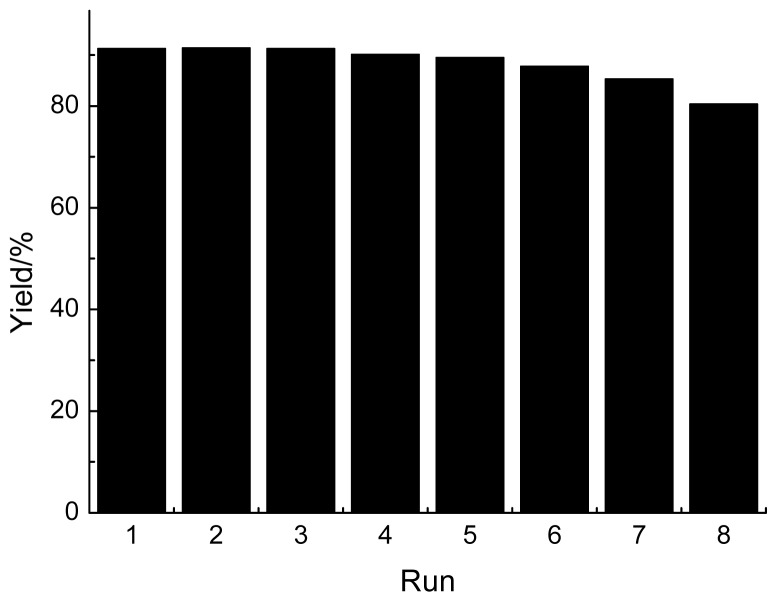
Reusability of the GBAIL-C_14_ catalyst. Reaction conditions: 1.0 mmol benzaldehyde, 1.0 mmol aniline, 1.0 mmol cyclohexanone, 0.050 mmol catalyst, 1.5 mL H_2_O, 5 h, 25 °C.

The reusability of the GBAIL-C_14_ catalyst in a model Mannich-type reaction was also investigated. After reaction, the products were isolated from the catalytic system by extraction with ethyl acetate. The combined aqueous extract (aqueous media containing the catalyst) was reused in subsequent runs without further purification. As shown in Figure 3, the catalyst can be reused at least seven times without any noticeable decrease in yield or reaction rate. The yields ranged from 91% to 85%. Compared with the reusability of traditional solvents and catalysts, the facile reusability of GBAIL-C_14_ is also an interesting property for environmental protection and economic reasons.

### 2.4. Catalytic Performance of GBAIL-C_14_ in Mannich Reactions in Water

To generalize our protocol, we investigated the reactions of various aromatic aldehydes, aromatic amines, and cyclohexanone in the presence of GBAIL-C_14_ in water at room temperature. The results are summarized in Table 3. In general, the reaction proceeded smoothly to produce the corresponding products in reasonable to good yields ranging from 87% to 94%. The order of reactivity of the amines was *p*-chloroaniline > aniline > *p*-anisidine > *o*-anisidine (entries 1–4), which indicates the importance of the electronic and steric nature of the amines. Aromatic aldehydes and anilines with either electron-withdrawing or electron-donating substituents could undergo the reaction, as shown previously in [29] (entries 5–8). Ten millimolar-scale reactions were also conducted without any difficulties. For example, the reaction of benzaldehyde (10 mmol), aniline (10 mmol), and cyclohexanone (10 mmol) in the presence of 5 mol % of the catalyst produced the desired product with 87% yield (entry 9).

## 3. Experimental Section

### 3.1. General

*N*,*N*-Dimethylethylenediamine was purchased from J&K Chemicals, Beijing, China. *n*-Butyl bromide, *n*-octyl bromide, *n*-decyl bromide, *n*-dodecyl bromide, *n*-tetradecyl bromide, and *p*-toluenesulfonic acid monohydrate were obtained from Aladdin Reagent Co., Shanghai, China and used without further purification. 1,3-Propanesulfonate (99%) was purchased from Energy Chemicals (Shanghai, China). *n*-Hexadecyl bromide and *n*-stearyl bromide were obtained from Chemxyz Reagent Co., Shanghai, China. Acetone, diethyl ether, ethanol, hexane, methanol, and sodium hydroxide were obtained from Tianjin Kemio Fine Chemical Institute, Tianjin, China and used directly.

FT-IR spectra were recorded using KBr tablets by a Bruker (Fällanden, Switzerland) Vector 22 spectrophotometer in the range of 400–4000 cm^−1. 1^H- and ^13^C-NMR spectra were obtained on a Bruker (Fällanden, Switzerland) AC-P400 spectrometer in D_2_O and CDCl_3_. Melting points were determined using an XT-4 (Keyi Electric Light Instrument Co., Ltd., Beijing, China) melting point apparatus and are reported uncorrected.

### 3.2. Synthesis of GBAILs

#### 3.2.1. Synthesis of Geminal Sulfobetaine-Type Zwitterion

Geminal sulfobetaine-type zwitterionic 1,2-bis[*N*-methyl-*N*-(3-sulfopropyl)-alkylammonium]ethane (where the carbon number of the alkyl was 4, 6, 8, 10, 12, 14, 16, or 18) was synthesized by reaction of *N*,*N*-dimethylethylenediamine with *n*-alkyl bromide, followed by 1,3-propanesulfonate based on the literature [35] with some modifications.

The procedure was based on the synthesis of 1,2-bis[*N*-methyl-*N*-(3-sulfopropyl)- tetradecylammonium]ethane. *n*-Tetradecyl bromide (0.042 mol, 11.65 g) was added to a stirred solution of *N*,*N*-dimethylethylenediamine (0.020 mol, 1.76 g) in ethanol containing NaOH (0.042 mol, 1.68 g), and the mixture was refluxed for over 40 h. The formed inorganic salt was removed and hydrochloric acid gas was injected into the residue dissolved in acetone, thereby producing *N*,*N*′-dimethyl-*N*,*N*′-ditetradecylethylenediamine hydrochloric acid salts as white solids. The obtained solid was washed with acetone and neutralized with 10% NaOH aqueous solution, and the product was extracted with hexane. White waxy solids were obtained after solvent evaporation.

A fourfold molar excess of 1,3-propanesulfonate was added to a stirred solution of *N*,*N*′-dimethyl-*N*,*N*′-ditetradecylethylenediamine in acetone, and the mixture was refluxed for 24 h. After the mixture had cooled to room temperature, the obtained precipitate was filtered, washed with acetone, recrystallized from mixtures of acetone and methanol, and dried under reduced pressure to produce 1,2-bis[*N*-methyl-*N*-(3-sulfopropyl)-tetradecylammonium]ethane as a white solid.

#### 3.2.2. GBAIL Preparation

Zwitterion acidification was accomplished by mixing the zwitterions with *p*-toluenesulfonic acid monohydrate based on a previously described method [29,36] with some modifications. Typically, the zwitterion (1 equiv.) and *p*-toluenesulfonic acid monohydrate (2 equiv.) were dissolved in 1 mL of ethanol. The mixture was magnetically stirred and heated to 80 °C in an open single-neck round-bottom flask until the solvent had evaporated. The mixture was then stirred for 5 h at 110 °C. After completion of the reaction, the viscous residue was dried under vacuum at 100 °C for 2 h. After cooling, a white solid product was obtained. The structures of intermediate materials and GBAILs were identified by IR, ^1^H-NMR, and ^13^C-NMR, and selected spectral data are listed in the supplementary information.

### 3.3. General Procedure for Mannich Reactions Catalyzed by GBAILs

In a typical procedure, a reaction tube was charged with the catalyst (1 to 7 mol %) in 1.5 mL of water. Aldehyde (1.0 mmol), ketone (1.0 mmol), and aniline (1.0 mmol) were added successively with stirring. The mixture was then stirred at 25 °C for the period of time listed in Table 2. After completion of the reaction (monitored by TLC), the mixture was extracted with ethyl acetate (2 mL) and the upper layer was removed via a pipette. Extraction was repeated three times, and the combined organic layers were concentrated and recrystallized from ethanol–acetone (*v*/*v* = 1:1) to produce the desired product. The reaction was repeated twice to obtain the average yield. The aqueous extract containing IL catalysts could be reused directly in subsequent runs without further purification.

## 4. Conclusions

In conclusion, several three-component Mannich-type reactions using aldehydes, amines, and cyclohexanone were efficiently catalyzed by GBAILs in water under mild conditions. The results showed that GBAIL-C_14_ provides excellent catalytic activity because of its ability to induce emulsion formation. The IL formed stable colloidal particles in the presence of the substrates in water; this colloid formation has an essential function in accelerating the reaction. The catalytic procedure was simple and the catalyst could be recycled seven times via a simple separation process without noticeable decreases in catalytic activity. Therefore, we believe that the work reported in this paper will have potential applications in green chemistry.

## Supplementary Information



## Figures and Tables

**Table 1.** Influence of the catalytic system on the Mannich reaction in water a.

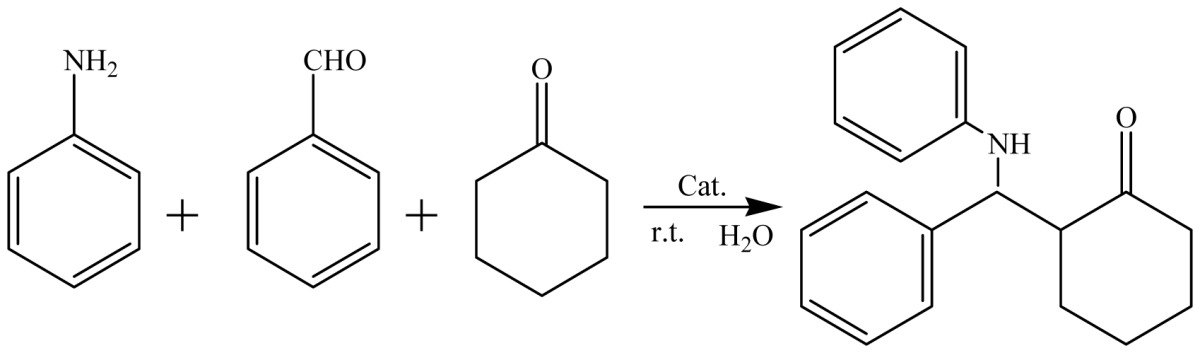		

Entry	Catalyst	Yield/% b
1	GBAIL-C_4_	64
2	GBAIL-C_8_	70
3	GBAIL-C_10_	76
4	GBAIL-C_12_	82
5	GBAIL-C_14_	88
6	GBAIL-C_16_	87
7	GBAIL-C_18_	85

a Reaction conditions: 1.0 mmol benzaldehyde, 1.0 mmol aniline, 1.0 mmol cyclohexanone, 0.050 mmol catalyst, 1.5 mL H_2_O, 4 h, 25 °C;

b Yield of the isolated product after purification by recrystallization.

**Table 2.** Effects of the amount of catalyst and reaction time on the Mannich reaction a.

Entry	Amount of catalyst/mmol	Time/h	Yield/% b
1	0.01	4	43
2	0.02	4	65
3	0.03	4	72
4	0.04	4	79
5	0.05	4	88
6	0.06	4	88
7	0.07	4	89
8	0.05	1	43
9	0.05	2	69
10	0.05	3	83
11	0.05	5	91
12	0.05	6	92
13	0.05	7	92
14	0.05	8	93

a Reaction conditions: 1.0 mmol benzaldehyde, 1.0 mmol aniline, 1.0 mmol cyclohexanone, 1.5 mL H_2_O, 25 °C;

b Yield of the isolated product after purification by recrystallization.

**Table 3.** Results of one-pot, three-component Mannich reactions with different substrates catalyzed by GBAIL-C_14_^a^.

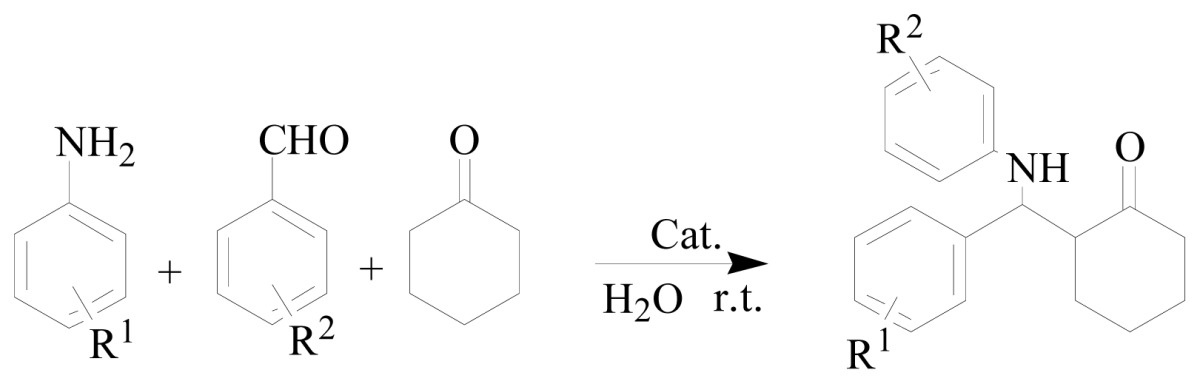			

Entry	R^1^	R^2^	Yield/% b
1	H	H	91
2	*p*-Cl	H	94
3	*p*-OMe	H	89
4	*o*-OMe	H	85
5	H	*o*-NO_2_	91
6	H	*p*-NO_2_	91
7	H	*o*-Cl	92
8	H	*p*-OMe	90
9	H	H	87 c

a Reaction conditions: 1.0 mmol aromatic aldehyde, 1.0 mmol aromatic amine, 1.0 mmol cyclohexanone, 0.050 mmol catalyst, 1.5 mL H_2_O, 5 h, 25 °C;

b Yield of the isolated product after purification by recrystallization;

c Reaction conditions: 10 mmol benzaldehyde, 10 mmol aniline, 10 mmol cyclohexanone, 0.50 mmol catalyst, 15 mL H_2_O, 5 h, 25 °C.
