# Supplementary material for: Geminal Brønsted Acid Ionic Liquids as Catalysts for the Mannich Reaction in Water
Source: Int J Mol Sci. 2014 May 15;15(5):8656–66. doi: 10.3390/ijms15058656 (PMC4057752; doi:10.3390/ijms15058656)
Supplement: Supplementary file 1 [file ijms-15-08656-s001.pdf]

Supplementary Information

(A) Spectral characterization data for catalysts and Mannich reaction products

N,N'-Dibutyl-N,N'-dimethylethylenediamine: Yield 54%; Colorless liquid; ^{1}H-NMR (400 MHz, $CDCl_3$, TMS): δ 0.91 (t, 6H, 2CH_3CH_2), 1.28–1.33 (m, 4H, 2$CH_3CH_2CH_2$), 1.41–1.47 (m, 4H, 2-$CH_2CH_2NCH_3(CH_2)$), 2.23 (s, 6H, 2-$CH_2NCH_2(CH_3)$), 2.30–2.34 (t, 4H, 2-$CH_2CH_2CH_2NCH_3(CH_2)$), 2.47 (t, 4H, $CH_2N(CH_3)CH_2CH_2N(CH_3)CH_2$; ^{13}C-NMR (100 MHz, $CDCl_3$): 14.11, 20.74, 29.46, 42.79, 55.62, 58.18.

N,N'-Dimethyl-N,N'-dioctylethylenediamine: Yield 60%; Colorless liquid; ^{1}H-NMR (400 MHz, $CDCl_3$, TMS): δ 0.87 (t, 6H, 2CH_3CH_2), 1.27–1.31 (m, 20H, 2$CH_3(CH_2)_5CH_2$), 1.45 (m, 4H, 2-$CH_2CH_2NCH_3(CH_2)$), 2.23 (s, 6H, 2-$CH_2NCH_2(CH_3)$), 2.31–2.35 (t, 4H, 2-$CH_2CH_2CH_2NCH_3(CH_2)$), 2.46 (t, 4H, $CH_2N(CH_3)CH_2CH_2N(CH_3)CH_2$; ^{13}C-NMR (100 MHz, $CDCl_3$): 14.11, 22.68, 27.29, 27.61, 27.50, 29.30, 29.60, 31.87, 42.78, 55.57, 58.48.

N,N'-Didecyl-N,N'-dimethylethylenediamine: Yield 63%; Colorless liquid; ^{1}H-NMR (400 MHz, $CDCl_3$, TMS): δ 0.87 (t, 6H, 2CH_3CH_2), 1.26–1.31 (m, 28H, 2$CH_3(CH_2)_7CH_2$), 1.45 (m, 4H, 2-$CH_2CH_2NCH_3(CH_2)$), 2.23 (s, 6H, 2-$CH_2NCH_2(CH_3)$), 2.31–2.35 (t, 4H, 2-$CH_2CH_2CH_2NCH_3(CH_2)$), 2.47 (t, 4H, $CH_2N(CH_3)CH_2CH_2N(CH_3)CH_2$; ^{13}C-NMR (100 MHz, $CDCl_3$): 14.07, 26.25, 27.25, 27.57, 29.31, 29.57, 29.60, 31.88, 42.72, 55.53, 58.43.

N,N'-Didodecyl-N,N'-dimethylethylenediamine: Yield 69%; Colorless liquid; ^{1}H-NMR (400 MHz, $CDCl_3$, TMS): δ 0.88 (t, 6H, 2CH_3CH_2), 1.25–1.31 (m, 36H, 2$CH_3(CH_2)_9CH_2$), 1.45 (m, 4H, 2-$CH_2CH_2NCH_3(CH_2)$), 2.23 (s, 6H, 2-$CH_2NCH_2(CH_3)$), 2.31–2.35 (t, 4H, 2-$CH_2CH_2CH_2NCH_3(CH_2)$), 2.46 (t, 4H, $CH_2N(CH_3)CH_2CH_2N(CH_3)CH_2$; ^{13}C-NMR (100 MHz, $CDCl_3$): 14.09, 22.67, 27.28, 27.59, 29.35, 29.63, 29.67, 31.91, 42.75, 55.56, 56.46.

N,N'-Dimethyl-N,N'-ditetradecylethylenediamine: Yield 74%; White solid; m.p. 36–37 °C ^{1}H-NMR (400 MHz, $CDCl_3$, TMS): δ 0.88 (t, 6H, 2CH_3CH_2), 1.25–1.27 (m, 44H, 2$CH_3(CH_2)_{11}CH_2$), 1.49 (m, 4H, 2-$CH_2CH_2NCH_3(CH_2)$), 2.29 (s, 6H, 2-$CH_2NCH_2(CH_3)$), 2.39–2.43 (t, 4H, 2-$CH_2CH_2CH_2NCH_3(CH_2)$), 2.57 (t, 4H, $CH_2N(CH_3)CH_2CH_2N(CH_3)CH_2$; ^{13}C-NMR (100 MHz, $CDCl_3$): 14.12, 22.70, 26.88, 27.50, 29.37, 29.57, 29.62, 29.63, 29.67, 29.69, 31.93, 42.42, 54.92, 58.19; FT-IR (KBr pellet, 4000–400 cm^{-1}) 718, 1032, 1381, 1470, 2453, 2852, 2921.

N,N'-Dihexadecyl-N,N'-dimethylethylenediamine: Yield 79%; White solid; m.p. 42–44 °C ^{1}H-NMR (400 MHz, $CDCl_3$, TMS): δ 0.88 (t, 6H, 2CH_3CH_2), 1.25–1.29 (m, 52H, 2$CH_3(CH_2)_{13}CH_2$), 1.46 (m, 4H, 2-$CH_2CH_2NCH_3(CH_2)$), 2.24 (s, 6H, 2-$CH_2NCH_2(CH_3)$), 2.33–2.37 (t, 4H, 2-$CH_2CH_2CH_2NCH_3(CH_2)$), 2.49 (t, 4H, $CH_2N(CH_3)CH_2CH_2N(CH_3)CH_2$; FT-IR (KBr pellet, 4000–400 cm^{-1}) 718, 1032, 1381, 1470, 2453, 2852, 2921; ^{13}C-NMR (100 MHz, $CDCl_3$): 14.13, 22.70, 27.19, 27.59, 29.37, 29.64, 29.67, 29.71, 31.94, 42.70, 54.41, 58.40; FT-IR (KBr pellet, 4000–400 cm^{-1}) 718, 1046, 1381, 1469, 2451, 2850, 2919.

N,N'-Dimethyl-N,N'-distearylethylenediamine: Yield 83%; White solid; m.p. 50–52 °C ^{1}H-NMR (400 MHz, $CDCl_3$, TMS): δ 0.88 (t, 6H, 2CH_3CH_2), 1.25–1.29 (m, 60H, 2$CH_3(CH_2)_{15}CH_2$),

1.46 (m, 4H, $2\text{-}\mathit{CH_2}CH_2NCH_3(CH_2)$), 2.24 (s, 6H, $2\text{-}CH_2NCH_2(\mathit{CH_3})$), 2.33–2.36 (t, 4H, $2\text{-}CH_2CH_2\mathit{CH_2}NCH_3(CH_2)$), 2.48 (t, 4H, $CH_2N(CH_3)\mathit{CH_2CH_2}N(CH_3)CH_2$); ^{13}C-NMR (100 MHz, $CDCl_3$): 14.11, 22.69, 27.21, 27.58, 29.36, 29.63, 29.66, 29.70, 31.93, 42.71, 55.45, 58.41; FT-IR (KBr pellet, 4000–400 cm^{-1}) 718, 1028, 1381, 1470, 2452, 2850, 2916.

1,2-bis(N-Methyl-N-(3-sulfopropyl)-butylammonium)ethane: Yield 89%; White solid; ^{1}H-NMR (D_2O, TMS) 0.81 (t, 6H, $2\mathit{CH_3}CH_2$), 1.20–1.24 (m, 4H, $2CH_3\mathit{CH_2}CH_2$), 1.60 (m, 4H, $2\text{-}\mathit{CH_2}CH_2N^+CH_3(CH_2)$), 2.09 (m, 4H, $2\text{-}N^+CH_2\mathit{CH_2}CH_2SO_3^-$), 2.81 (t, 4H, $2\text{-}N^+CH_2CH_2\mathit{CH_2}SO_3^-$), 2.86 (s, 6H, $2\text{-}CH_2N^+CH_2(CH_2)(\mathit{CH_3})$), 3.11 (t, 4H, $2\text{-}CH_2CH_2\mathit{CH_2}$ $N^+CH_2(CH_2)(CH_3)$), 3.26–3.28 (t, 4H, $2\text{-}N^+\mathit{CH_2}CH_2CH_2SO_3^-$), 3.53 (t, 4H, $N^+\mathit{CH_2CH_2}N^+$); ^{13}C-NMR (100 MHz, D_2O): 12.68, 17.73, 18.95, 23.52, 25.37, 26.85, 40.09, 49.46, 60.14; FT-IR (KBr pellet, 4000–400 cm^{-1}) 529, 606, 734, 1042, 1201, 1474, 1644, 2960.

1,2-bis(N-Methyl-N-(3-sulfopropyl)-octylammonium)ethane: Yield 84%; White solid; ^{1}H-NMR (D_2O, TMS) 0.76 (t,6H, $2\mathit{CH_3}CH_2$), 1.18–1.28 (m, 20H, $2CH_3(\mathit{CH_2})_5CH_2$), 1.69 (m, 4H, $2\text{-}\mathit{CH_2}CH_2N^+CH_3(CH_2)$), 2.14 (m, 4H, $2\text{-}N^+CH_2\mathit{CH_2}CH_2SO_3^-$), 2.90 (t, 4H, $2\text{-}N^+CH_2CH_2\mathit{CH_2}SO_3^-$), 3.11 (s, 6H, $2\text{-}CH_2N^+CH_2(CH_2)(\mathit{CH_3})$), 3.35 (t, 4H, $2\text{-}CH_2CH_2\mathit{CH_2}$ $N^+CH_2(CH_2)(CH_3)$), 3.48–3.52 (t, 4H, $2\text{-}N^+\mathit{CH_2}CH_2CH_2SO_3^-$), 3.81 (t, 4H, $N^+\mathit{CH_2CH_2}N^+$); ^{13}C-NMR (100 MHz, D_2O): 13.35, 17.90, 21.66, 21.95, 25.27, 28.14, 28.15, 30.92, 46.74, 48.65, 53.06, 60.41, 60.52, 62.81, 62.89; FT-IR (KBr pellet, 4000–400 cm^{-1}) 529, 608, 732, 1042, 1200, 1470, 1642, 2927.

1,2-bis(N-Methyl-N-(3-sulfopropyl)-decylammonium)ethane: Yield 87%; White solid; ^{1}H-NMR (DMSO, TMS) 0.86 (t,6H, $2\mathit{CH_3}CH_2$), 1.26–1.30 (m, 28H, $2CH_3(\mathit{CH_2})_7CH_2$), 1.73 (m, 4H, $2\text{-}\mathit{CH_2}CH_2N^+CH_3(CH_2)$), 2.02 (m, 4H, $2\text{-}N^+CH_2\mathit{CH_2}CH_2SO_3^-$), 3.09 (t, 4H, $2\text{-}N^+CH_2CH_2\mathit{CH_2}SO_3^-$), 3.30 (s, 6H, $2\text{-}CH_2N^+CH_2(CH_2)(\mathit{CH_3})$), 3.40 (t, 4H, $2\text{-}CH_2CH_2\mathit{CH_2}$ $N^+CH_2(CH_2)(CH_3)$), 3.46–3.50 (t, 4H, $2\text{-}N^+\mathit{CH_2}CH_2CH_2SO_3^-$), 3.91 (t, 4H, $N^+\mathit{CH_2CH_2}N^+$); ^{13}C-NMR (100 MHz, DMSO): 13.92, 18.39, 21. 35, 22.06, 25.81, 28.51, 28.65, 28.91, 31.26, 46.78, 48.13, 59.68, 62.35.

1,2-bis(N-Methyl-N-(3-sulfopropyl)-dodecylammonium)ethane: Yield 76%; White solid; ^{1}H-NMR (DMSO, TMS) 0.85 (t,6H, $2\mathit{CH_3}CH_2$), 1.25 (m, 36H, $2CH_3(\mathit{CH_2})_9CH_2$), 1.72 (m, 4H, $2\text{-}\mathit{CH_2}CH_2N^+CH_3(CH_2)$), 2.00 (m, 4H, $2\text{-}N^+CH_2\mathit{CH_2}CH_2SO_3^-$), 3.09 (t, 4H, $2\text{-}N^+CH_2CH_2\mathit{CH_2}SO_3^-$), 3.30 (s, 6H, $2\text{-}CH_2N^+CH_2(CH_2)(\mathit{CH_3})$), 3.33 (t, 4H, $2\text{-}CH_2CH_2\mathit{CH_2}$ $N^+CH_2(CH_2)(CH_3)$), 3.49 (t, 4H, $2\text{-}N^+\mathit{CH_2}CH_2CH_2SO_3^-$), 3.86 (t, 4H, $N^+\mathit{CH_2CH_2}N^+$); ^{13}C-NMR (100 MHz, $CDCl_3$): 14.15, 15.26, 22.71, 23.62, 26.20, 26.46, 28.61, 29.35, 29.42, 29.52, 29.61, 29.73, 29.81, 31.93, 32.59, 44.03, 66.05; FT-IR (KBr pellet, 4000–400 cm^{-1}) 529, 604, 732, 1040, 1199, 1469, 1645, 2925.

1,2-bis(N-Methyl-N-(3-sulfopropyl)-tetradecylammonium)ethane: Yield 89%; White solid; ^{1}H-NMR ($CDCl_3$, TMS) 0.89 (t, 6H, $2\mathit{CH_3}CH_2$), 1.18–1.25 (m, 44H, $2CH_3(\mathit{CH_2})_{11}CH_2$), 1.73 (m, 4H, $2\text{-}\mathit{CH_2}CH_2N^+CH_3(CH_2)$), 2.18 (m, 4H, $2\text{-}N^+CH_2\mathit{CH_2}CH_2SO_3^-$), 2.91 (t, 4H, $2\text{-}N^+CH_2CH_2\mathit{CH_2}SO_3^-$), 2.96 (s, 6H, $2\text{-}CH_2N^+CH_2(CH_2)(\mathit{CH_3})$), 3.26 (t, 4H, $2\text{-}CH_2CH_2\mathit{CH_2}$ $N^+CH_2(CH_2)(CH_3)$), 3.40–3.47 (t, 4H, $2\text{-}N^+\mathit{CH_2}CH_2CH_2SO_3^-$), 3.59 (t, 4H, $N^+\mathit{CH_2CH_2}N^+$); ^{13}C-NMR (100 MHz, $CDCl_3$): 14.14, 15.25, 19.75, 22.70, 23.61, 26.19, 26.73, 28.28, 29.36, 29.52, 29.61, 29.66, 29.80, 31.92, 32.43, 44.01, 47.74, 48.54, 66.06.

1,2-bis(N-Methyl-N-(3-sulfopropyl)-hexadecylammonium)ethane: Yield 90%; White solid; ^{1}H-NMR ($CDCl_3$, TMS) 0.88 (t, 6H, $2CH_3CH_2$), 1.25–1.31 (m, 52H, $2CH_3(CH_2)_{13}CH_2$), 1.74 (m, 4H, $2\text{-}CH_2CH_2N^+CH_3(CH_2)$), 2.21 (m, 4H, $2\text{-}N^+CH_2CH_2CH_2SO_3^-$), 2.92 (t, 4H, $2\text{-}N^+CH_2CH_2CH_2SO_3^-$), 3.01 (s, 6H, $2\text{-}CH_2N^+CH_2(CH_2)(CH_3)$), 3.31 (t, 4H, $2\text{-}CH_2CH_2CH_2$ $N^+CH_2(CH_2)(CH_3)$), 3.40–3.47 (t, 4H, $2\text{-}N^+CH_2CH_2CH_2SO_3^-$), 3.68 (t, 4H, $N^+CH_2CH_2N^+$); ^{13}C-NMR (100 MHz, $CDCl_3$): 14.15, 18.79, 22.53, 22.71, 23.62, 24.17, 26.20, 26.61, 28.65, 29.16, 29.41, 29.47, 29.53, 29.72, 29.79, 31.95, 32.57, 44.03, 47.57, 48.58, 66.05; FT-IR (KBr pellet, 4000–400 cm^{-1}) 529, 606, 730, 1042, 1198, 1469, 1643, 2853, 2920.

1,2-bis(N-Methyl-N-(3-sulfopropyl)-steraylammonium)ethane: Yield 92%; White solid; ^{1}H-NMR ($CDCl_3$, TMS) 0.88 (t, 6H, $2CH_3CH_2$), 1.25–1.31 (m, 60H, $2CH_3(CH_2)_{15}CH_2$), 1.74 (m, 4H, $2\text{-}CH_2CH_2N^+CH_3(CH_2)$), 2.27 (m, 4H, $2\text{-}N^+CH_2CH_2CH_2SO_3^-$), 2.96 (t, 4H, $2\text{-}N^+CH_2CH_2CH_2SO_3^-$), 3.31 (s, 6H, $2\text{-}CH_2N^+CH_2(CH_2)(CH_3)$), 3.26 (t, 4H, $2\text{-}CH_2CH_2CH_2$ $N^+CH_2(CH_2)(CH_3)$), 3.40–3.45 (t, 4H, $2\text{-}N^+CH_2CH_2CH_2SO_3^-$), 3.68 (t, 4H, $N^+CH_2CH_2N^+$); FT-IR (KBr pellet, 4000–400 cm^{-1}) 529, 607, 726, 1038, 1207, 1469, 1648, 2851, 2920.

GBAIL-C_4: 1H-NMR (D_2O, TMS) 0.75 (t, 6H, $2CH_3CH_2$), 1.12–1.16 (m, 4H, $2CH_3CH_2CH_2$), 1.52 (m, 4H, $2\text{-}CH_2CH_2N^+CH_3(CH_2)$), 2.03 (m, 4H, $2\text{-}N^+CH_2CH_2CH_2SO_3^-$), 2.14 (s, 3H, Ar-$CH_3$), 2.72 (t, 4H, $2\text{-}N^+CH_2CH_2CH_2SO_3^-$), 2.76 (s, 6H, $2\text{-}CH_2N^+CH_2(CH_2)(CH_3)$), 2.97 (t, 4H, $2\text{-}CH_2CH_2CH_2$ $N^+CH_2(CH_2)(CH_3)$), 3.18–3.20 (t, 4H, $2\text{-}N^+CH_2CH_2CH_2SO_3^-$), 3.35 (t, 4H, $N^+CH_2CH_2N^+$), 7.13 (d, 2H, Ar-*H*), 7.44 (d, 2H, Ar-*H*); ^{13}C-NMR (100 MHz, $CDCl_3$): 12.62, 17.17, 19.32, 20.43, 39.85, 46.61, 48.45, 60.58, 62.51, 125.29, 129.42, 139.23, 142.43.

GBAIL-C_8: ^{1}H-NMR (D_2O, TMS) 0.75 (t, 6H, $2CH_3CH_2$), 1.02–1.15 (m, 20H, $2CH_3(CH_2)_5CH_2$), 1.50 (m, 4H, $2\text{-}CH_2CH_2N^+CH_3(CH_2)$), 2.10 (m, 4H, $2\text{-}N^+CH_2CH_2CH_2SO_3^-$), 2.22 (s, 3H, Ar-$CH_3$), 2.85 (t, 4H, $2\text{-}N^+CH_2CH_2CH_2SO_3^-$), 3.07 (s, 6H, $2\text{-}CH_2N^+CH_2(CH_2)(CH_3)$), 3.26 (t, 4H, $2\text{-}CH_2CH_2CH_2N^+CH_2(CH_2)(CH_3)$), 3.45 (t, 4H, $2\text{-}N^+CH_2CH_2CH_2SO_3^-$), 3.49 (t, 4H, $N^+CH_2CH_2N^+$), 7.17 (d, 2H, Ar-*H*), 7.56 (d, 2H, Ar-*H*); ^{13}C-NMR (100 MHz, D_2O): 13.78, 16.74. 19.96, 20.54, 22.12, 22.50, 25.68, 28.97, 29.01, 31.70, 46.94, 46.98, 48.69, 52.32, 57.38, 61.49, 61.97, 125.42, 129.22, 140.32, 141.53.

GBAIL-C_{10}: ^{1}H-NMR (D_2O, TMS) 0.78 (t, 6H, $2CH_3CH_2$), 1.02–1.13 (m, 28H, $2CH_3(CH_2)_7CH_2$), 2.11 (m, 4H, $2\text{-}CH_2CH_2N^+CH_3(CH_2)$), 2.04 (m, 4H, $2\text{-}N^+CH_2CH_2CH_2SO_3^-$), 2.30 (s, 3H, Ar-$CH_3$), 2.85 (t, 4H, $2\text{-}N^+CH_2CH_2CH_2SO_3^-$), 3.08 (s, 6H, $2\text{-}CH_2N^+CH_2(CH_2)(CH_3)$), 3.47 (t, 4H, $2\text{-}CH_2CH_2CH_2$ $N^+CH_2(CH_2)(CH_3)$), 3.41–3.44 (t, 4H, $2\text{-}N^+CH_2CH_2CH_2SO_3^-$), 3.50 (t, 4H, $N^+CH_2CH_2N^+$), 7.16 (d, 2H, Ar-*H*), 7.56 (d, 2H, Ar-*H*); ^{13}C-NMR (100 MHz, D_2O): 13.83, 18.03, 20.60, 22.28, 22.77, 25.87, 29.40–29.97, 32.21, 47.05, 48.75–48.89, 61.09, 61.93, 125.47, 129.18, 140.90.

GBAIL-C_{12}: ^{1}H-NMR ($CDCl_3$, TMS) 0.89 (t, 6H, $2CH_3CH_2$), 1.08–1.27 (m, 36H, $2CH_3(CH_2)_9CH_2$), 1.60 (m, 4H, $2\text{-}CH_2CH_2N^+CH_3(CH_2)$), 2.31 (m, 4H, $2\text{-}N^+CH_2CH_2CH_2SO_3^-$), 2.31 (s, 3H, Ar-$CH_3$), 3.17 (t, 4H, $2\text{-}N^+$ $CH_2CH_2CH_2SO_3^-$), 3.27 (s, 6H, $2\text{-}CH_2N^+CH_2(CH_2)(CH_3)$), (t, 3.37(4H, $2\text{-}CH_2CH_2CH_2$ $N^+CH_2(CH_2)(CH_3)$), 3.64 (t, 4H, $2\text{-}N^+CH_2CH_2CH_2SO_3^-$), 4.00 (t, 4H, $N^+CH_2CH_2N^+$), 7.14 (d, 2H, Ar-*H*), 7.66 (d, 2H, Ar-*H*), 12.34 (m 1H, SO_3H); ^{13}C-NMR (100 MHz, $CDCl_3$): 14.15,

21.38, 22.71, 23.60, 26.09, 26.22, 27.55, 29.15, 29.27, 29.44, 29.62, 29.71, 29.80, 31.65, 31.93, 44.56, 49.95, 126.11, 129.33, 138.92, 142.00.

GBAIL-C_{14}: ^{1}H-NMR ($CDCl_3$, TMS) 0.86 (t, 6H, 2*CH_3*CH_2), 1.06–1.09 (m, 44H, $2CH_3(CH_2)_{11}CH_2$), 1.55 (m, 4H, 2-*CH_2*$CH_2N^+CH_3(CH_2)$)), 2.25 (m, 4H, 2-N^+CH_2*CH_2*$CH_2SO_3^-$), 2.32 (s, 3H, Ar-CH_3), 2.84 (t, 4H, 2-N^+ CH_2CH_2*CH_2*SO_3^-), 2.95 (s, 6H, 2-$CH_2N^+CH_2(CH_2)$(*CH_3*)), 3.18 (t, 4H, 2-CH_2CH_2*CH_2* $N^+CH_2(CH_2)(CH_3)$)), 3.41–3.42 (t, 4H, 2-N^+*CH_2*$CH_2CH_2SO_3^-$), 3.50 (t, 4H, N^+*CH_2CH_2*N^+), 7.15 (d, 2H, Ar-*H*), 7.67 (d, 2H, Ar-*H*), 12.48 (m 1H, SO_3*H*); ^{13}C-NMR (100 MHz, $CDCl_3$): 13.92, 18.81, 20.75, 22.05, 28.68, 28.99, 29.04, 31.26, 38.82, 40.08, 55.98, 125.45, 128.11, 137.85, 145.25.

GBAIL-C_{16}: ^{1}H-NMR ($CDCl_3$, TMS) 0.88 (t, 6H, 2*CH_3*CH_2), 1.20–1.29 (m, 52H, $2CH_3(CH_2)_{13}CH_2$), 1.58 (m, 4H, 2-*CH_2*$CH_2N^+CH_3(CH_2)$)), 2.26 (m, 4H, 2-N^+CH_2*CH_2*$CH_2SO_3^-$), 2.41 (s, 3H, Ar-CH_3), 2.93 (t, 4H, 2-$N^+CH_2CH_2$*CH_2*SO_3^-), 3.01 (s, 6H, 2-$CH_2N^+CH_2(CH_2)$(*CH_3*)), 3.31 (t, 4H, 2-CH_2CH_2*CH_2* $N^+CH_2(CH_2)(CH_3)$)), 3.40–3.47 (t, 4H, 2-N^+*CH_2*$CH_2CH_2SO_3^-$), 3.68 (t, 4H, N^+*CH_2CH_2*N^+), 7.17 (d, 2H, Ar-*H*), 7.70 (d, 2H, Ar-*H*), 12.85 (m 1H, SO_3*H*); ^{13}C-NMR (100 MHz, $CDCl_3$): 14.13, 21.33, 22.70, 26.36, 29.24, 29.40, 29.57, 29.71, 29.81, 29.84, 31.95, 125.91, 129.12, 140.90; ^{13}C-NMR (100 MHz, $CDCl_3$): 14.13, 21.35, 22.70, 29.24, 29.39, 29.58, 29.69, 29.76, 29.78, 29.18, 31.94, 125.92, 129.11, 140.89, 140.99.

2-(Phenyl(phenylamino) methyl) cyclohexanone (Table 3, entry 1): ^{1}H-NMR ($CDCl_3$), 1.65–1.71 (m, 2H), 1.79–1.92 (m, 4H), 2.31–2.40 (m, 1H), 2.42–2.43 (m, 1H), 2.74–2.76 (m, 1H), 4.60 (d, 1H), 4.62 (s, br, 1H), 6.52–6.54 (m, 2H), 6.59–6.63 (m, 1H), 7.03–7.07 (m, 2H), 7.18–7.23 (m, 1H), 7.26–7.30 (m, 2H), 7.35–7.37 (m, 2H); ^{13}C-NMR (100 MHz, $CDCl_3$): 23.67, 27.91, 31.92, 41.80, 57.45, 58.08, 113.70, 117.61, 127.20, 127.30, 128.49, 141.64, 147.13, 212.90.

2-(Phenyl(4-chlorophenylamino)methyl) cyclohexanone (Table 3, entry 2): ^{1}H-NMR ($CDCl_3$), 1.63–1.69 (m, 2H), 1.79–1.96 (m, H), 2.33–2.45 (m, 2H), 2.83–2.84 (m, 1H), 4.53 (d, 1H), 6.45–6.49 (m, 2H), 6.99–7.02 (m, 2H), 7.20–7.27 (m, 7H); ^{13}C-NMR (100 MHz, $CDCl_3$): 23.86, 23.89, 24.85, 41.99, 56.39, 57.14, 115.27, 122.21, 127.30, 128.34-128.91, 129.24, 144.35, 150.48.

2-(4-Nitrophenyl(phenylamino)methyl) cyclohexanone (Table 3, entry 6): ^{1}H-NMR ($CDCl_3$), 1.72–1.79 (m, 2H), 1.92–2.06 (m, 4H), 2.32–2.42 (m, 2H), 2.83–2.86 (m, 1H), 4.70 (d, 1H), 4.86 (s, br, 1H), 6.48–6.51 (m, 2H), 6.53–6.69 (m, 1H), 7.06–7.10 (m, 2H), 7.52–7.58 (m, 2H), 8.13–8.15 (m, 2H); ^{13}C-NMR (100 MHz, $CDCl_3$): 23.90, 28.09, 33.80, 40.61, 41.98, 55.37, 55.99, 113.96, 118.33, 127.97, 128.92, 129.29, 138.77, 146.70.

2-(2-Chlorophenyl(phenylamino)methyl) cyclohexanone (Table 3, entry 7): ^{1}H-NMR ($CDCl_3$), 1.73–1.79 (m, 2H), 1.94–2.08 (m, 4H), 2.29–2.38 (m, 2H), 2.91–2.95 (m, 1H), 4.90 (d, 1H), 5.41 (s, br, 1H), 6.51–6.53 (m, 2H), 6.61–6.64 (m, 1H), 7.05–7.19 (m, 4H), 7.24–7.33 (m, 1H), 7.55–7.57 (m, 1H); ^{13}C-NMR (100 MHz, $CDCl_3$): 24.80, 28.09, 32.66, 42.83, 55.48, 113.48, 117.71, 127.07, 128.26, 129.00, 129.17, 129.28, 133.33, 139.02, 146.71, 213.19.

(B) Copies of NMR spectra for intermediate materials and GBAILs

Figure S1. Copies of NMR spectra for *N*,*N'*-dimethyl-*N*,*N'*-dialkylethylenediamine.

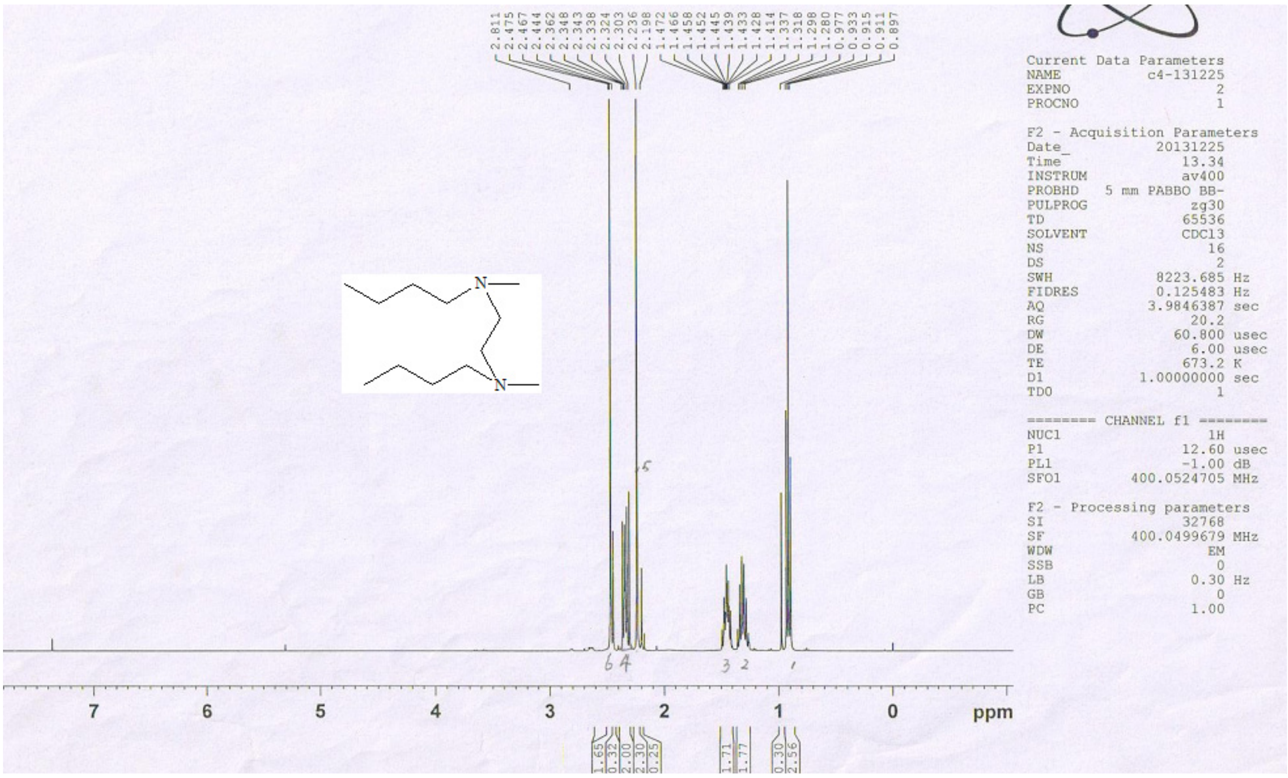

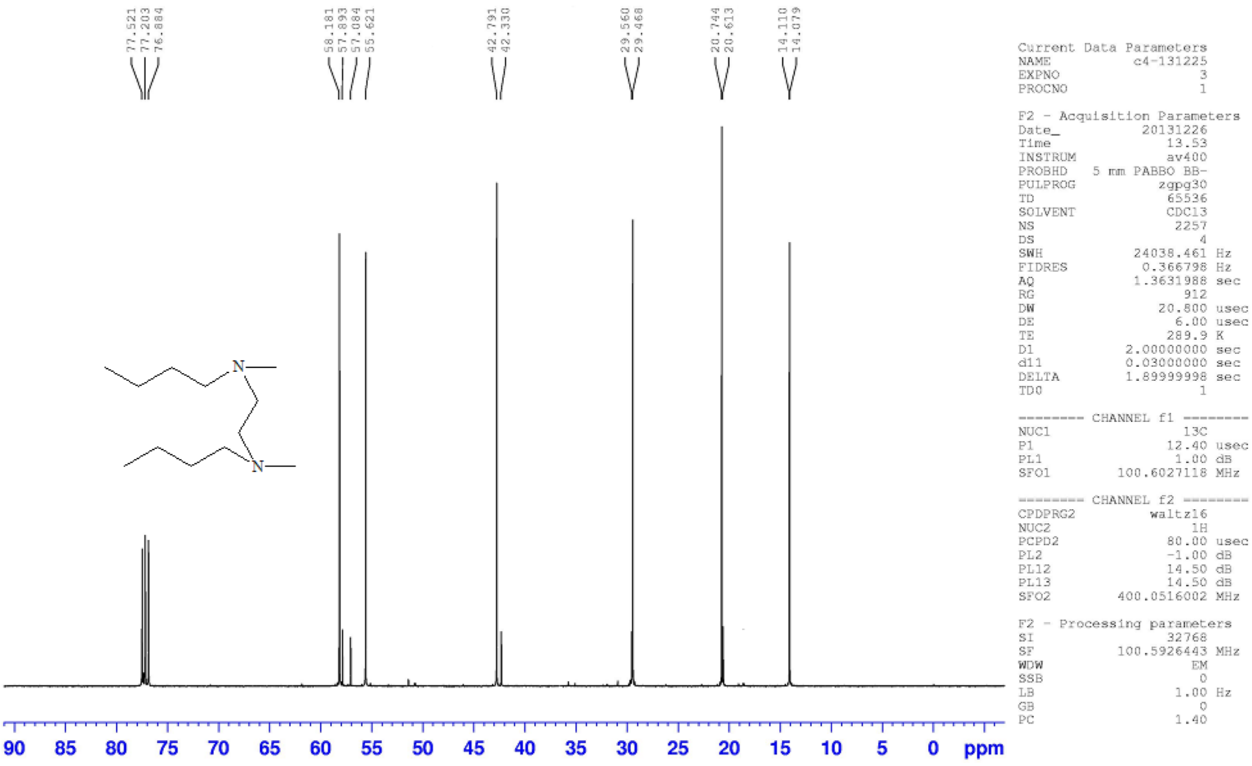

Figure S1. *Cont.*

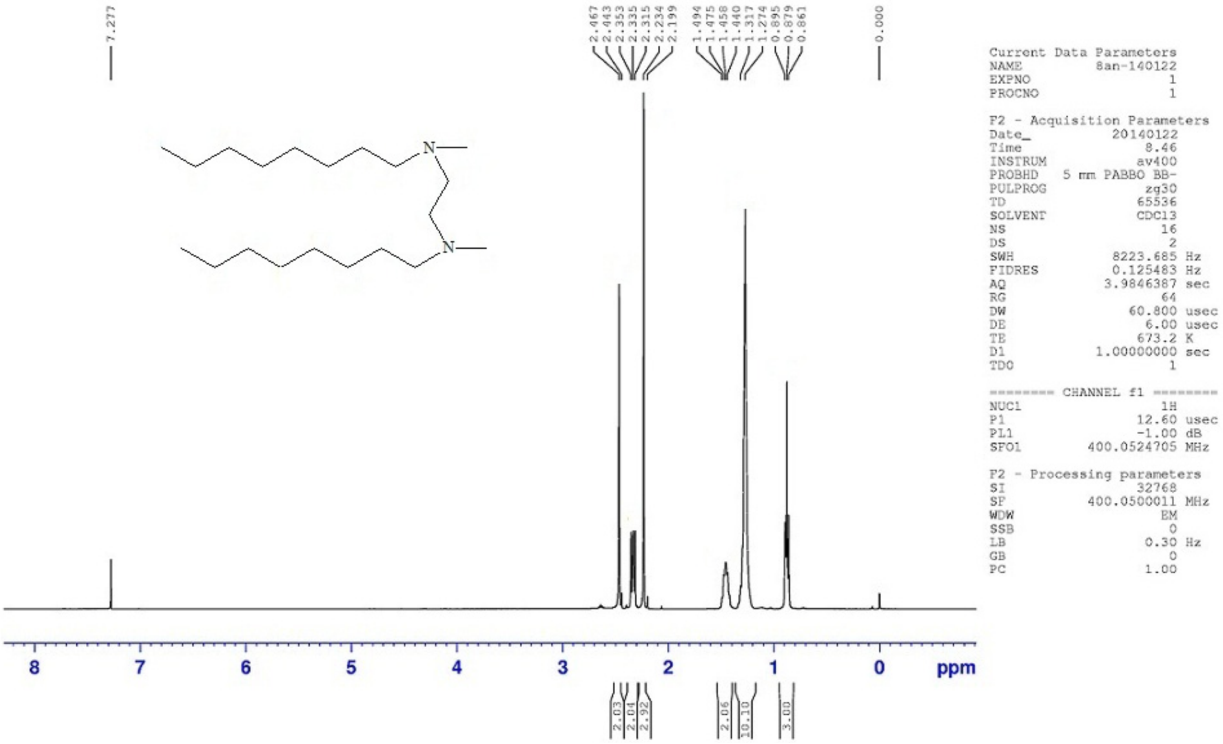

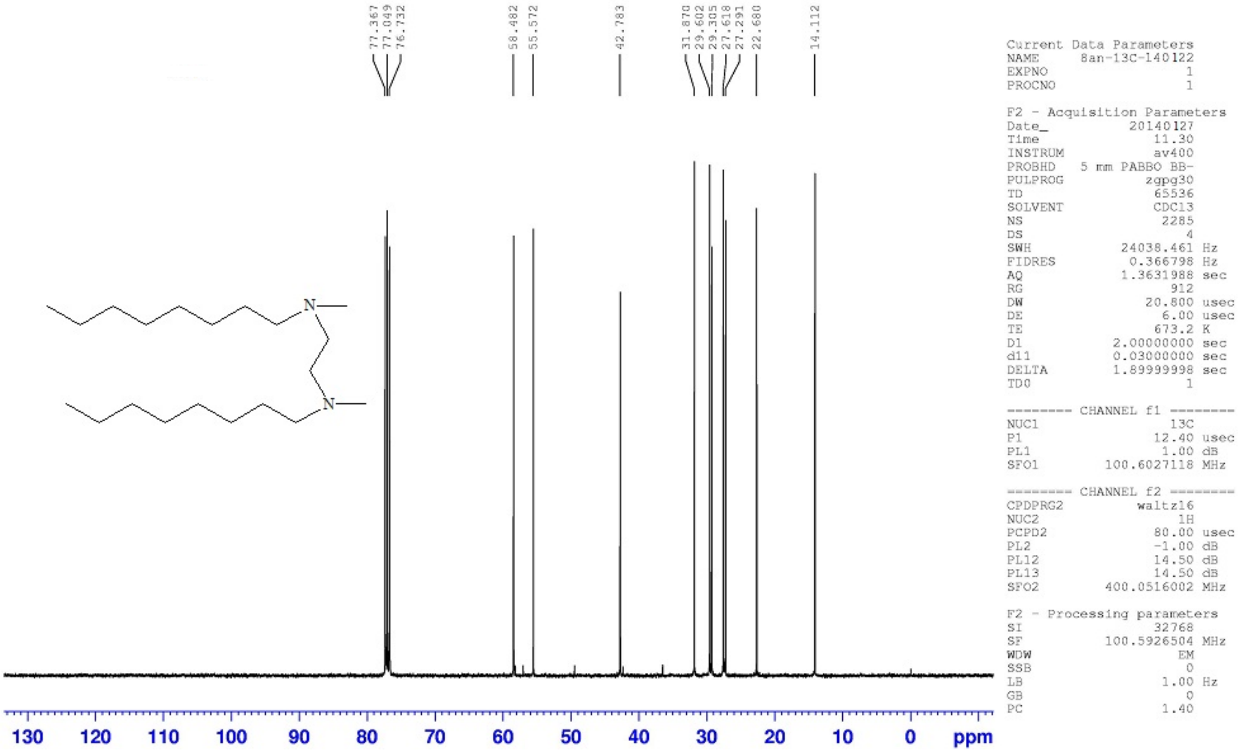

Figure S1. *Cont.*

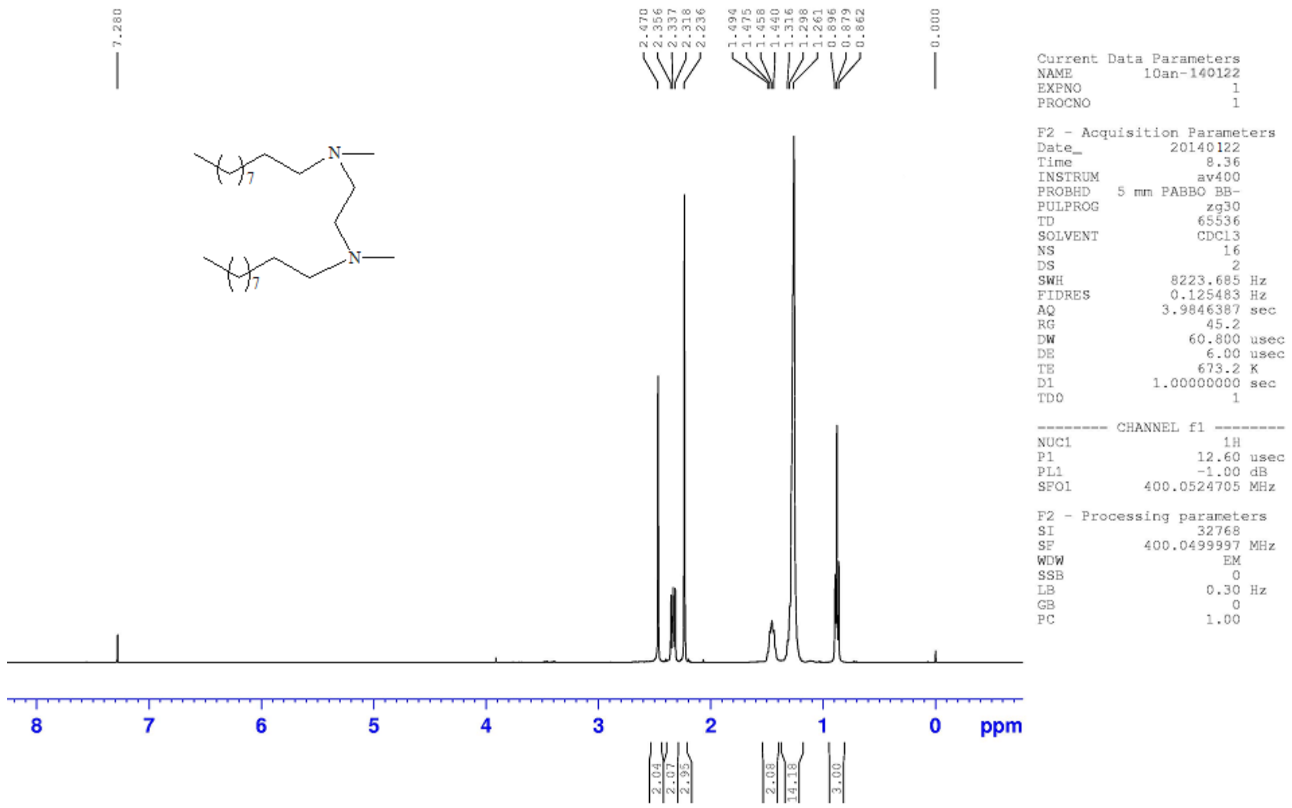

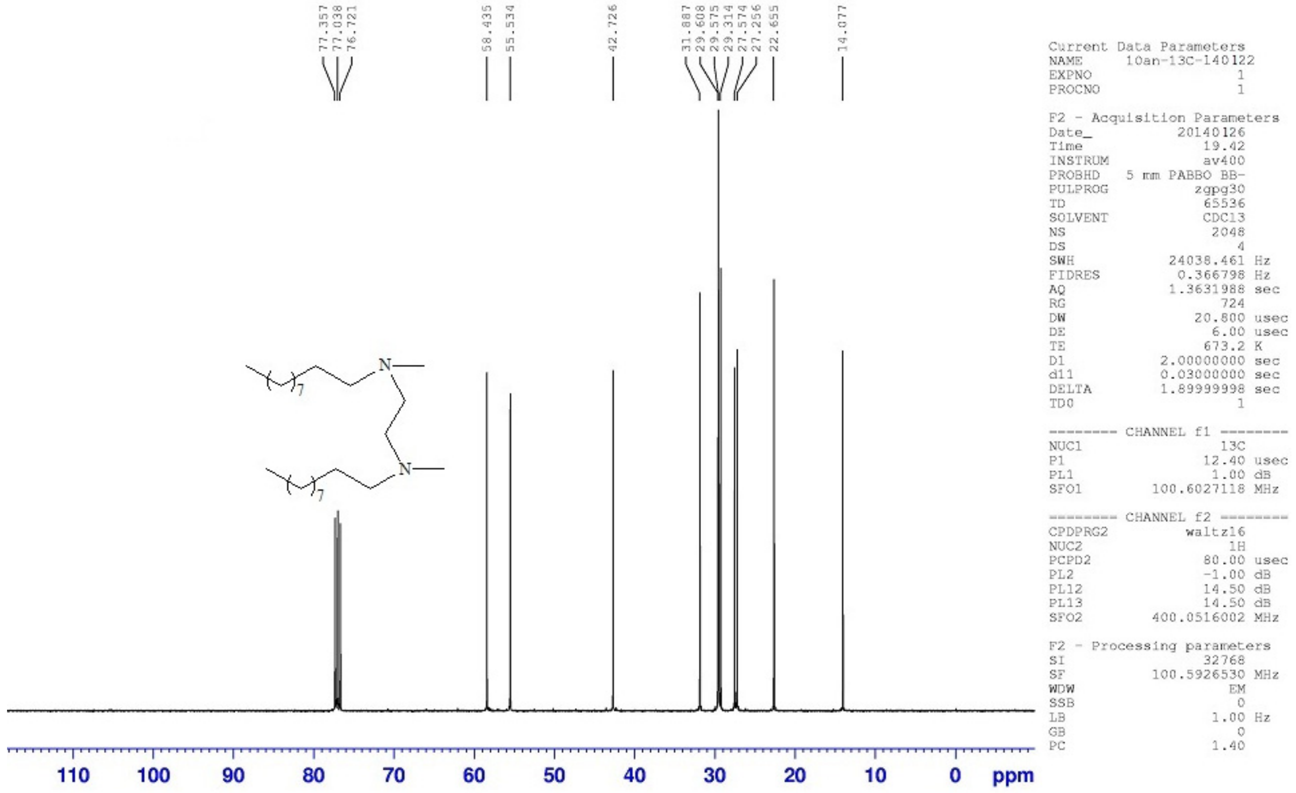

Figure S1. *Cont.*

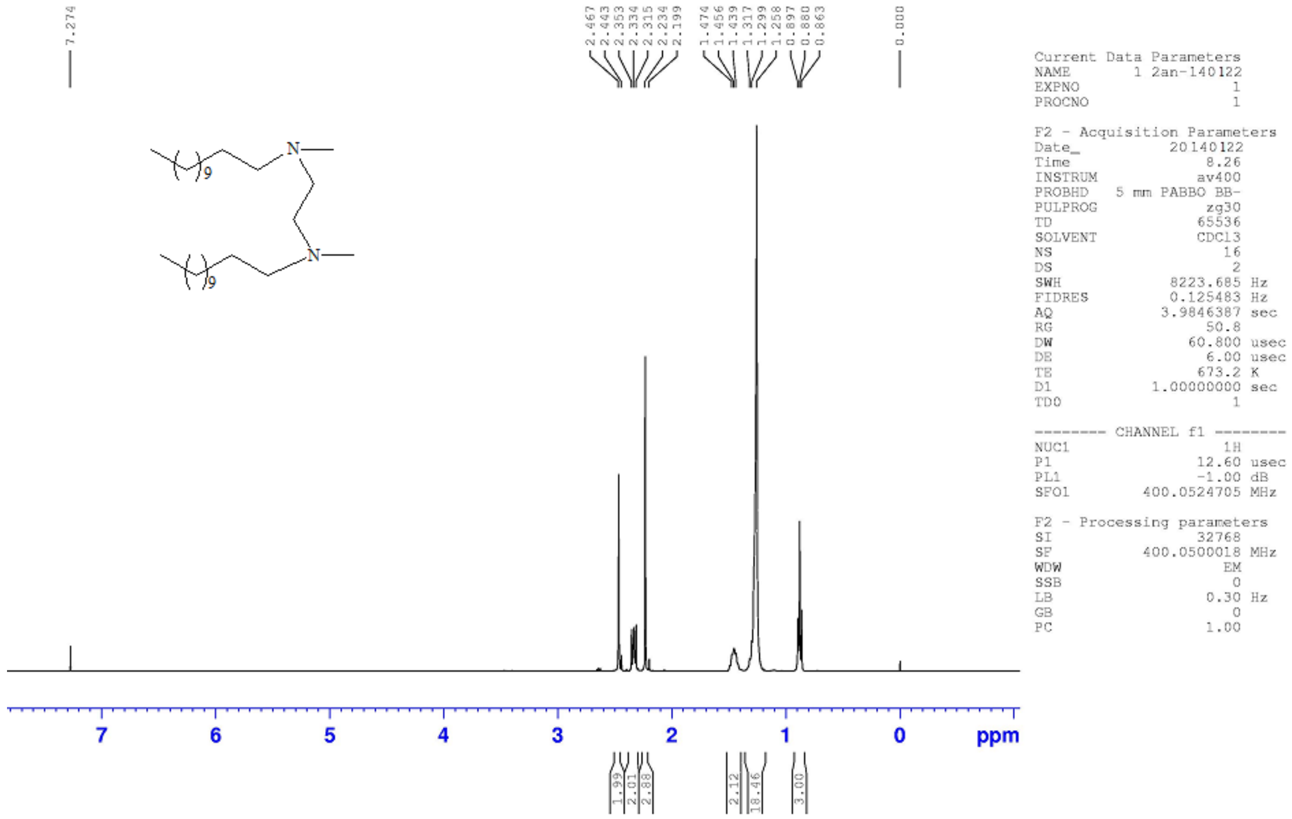

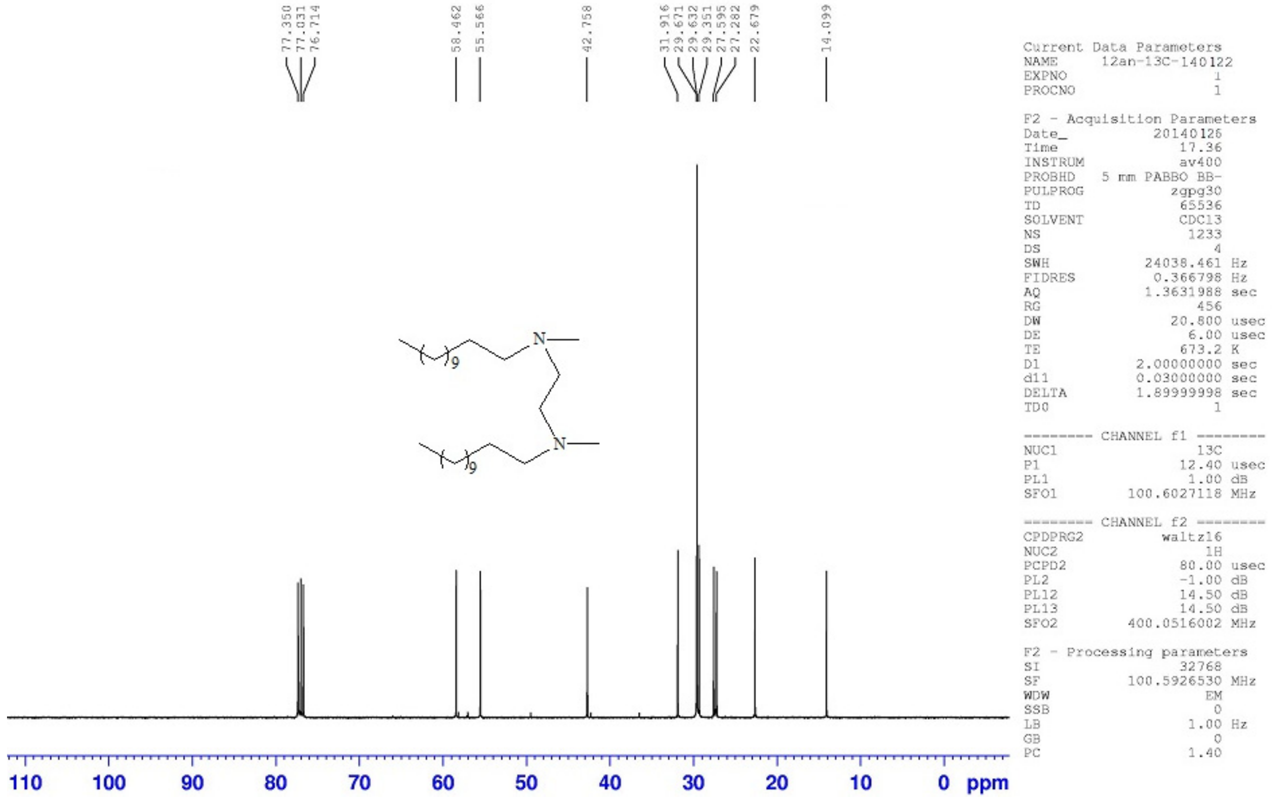

Figure S1. *Cont.*

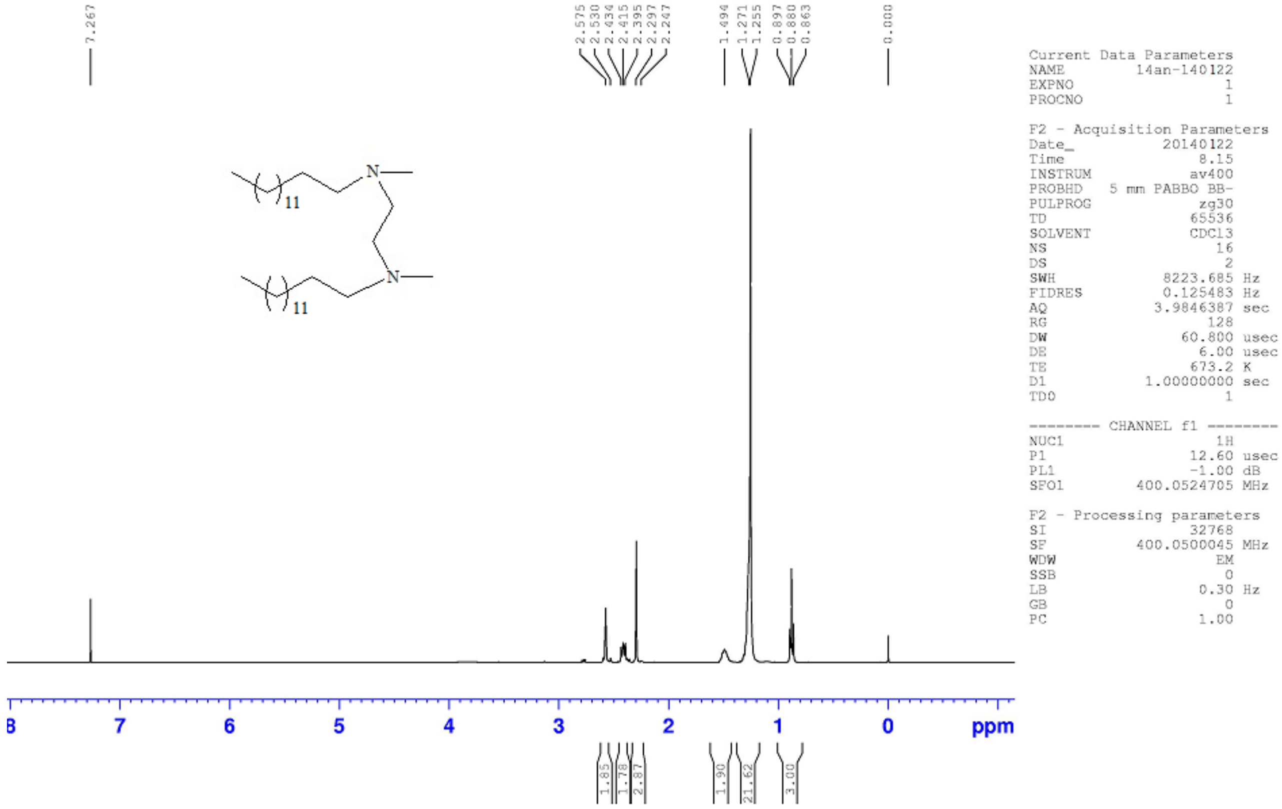

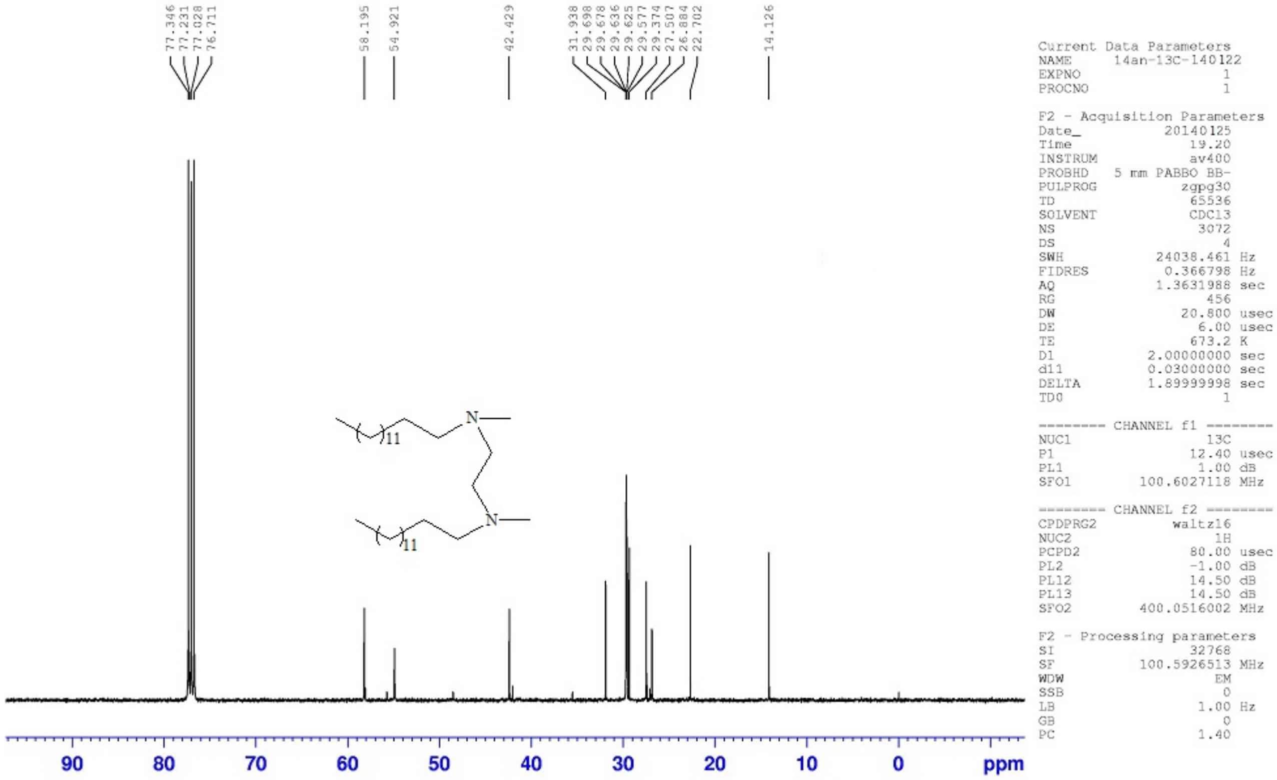

Figure S1. *Cont.*

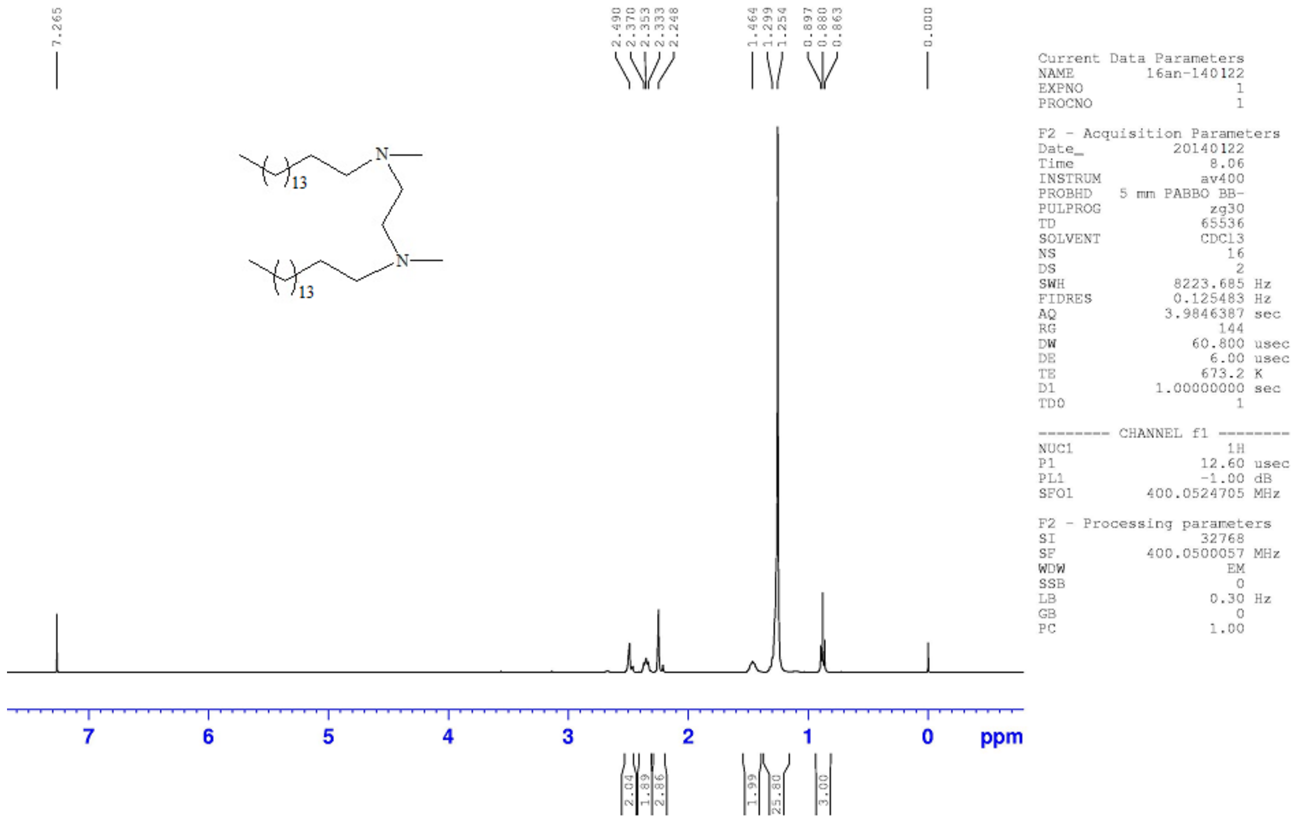

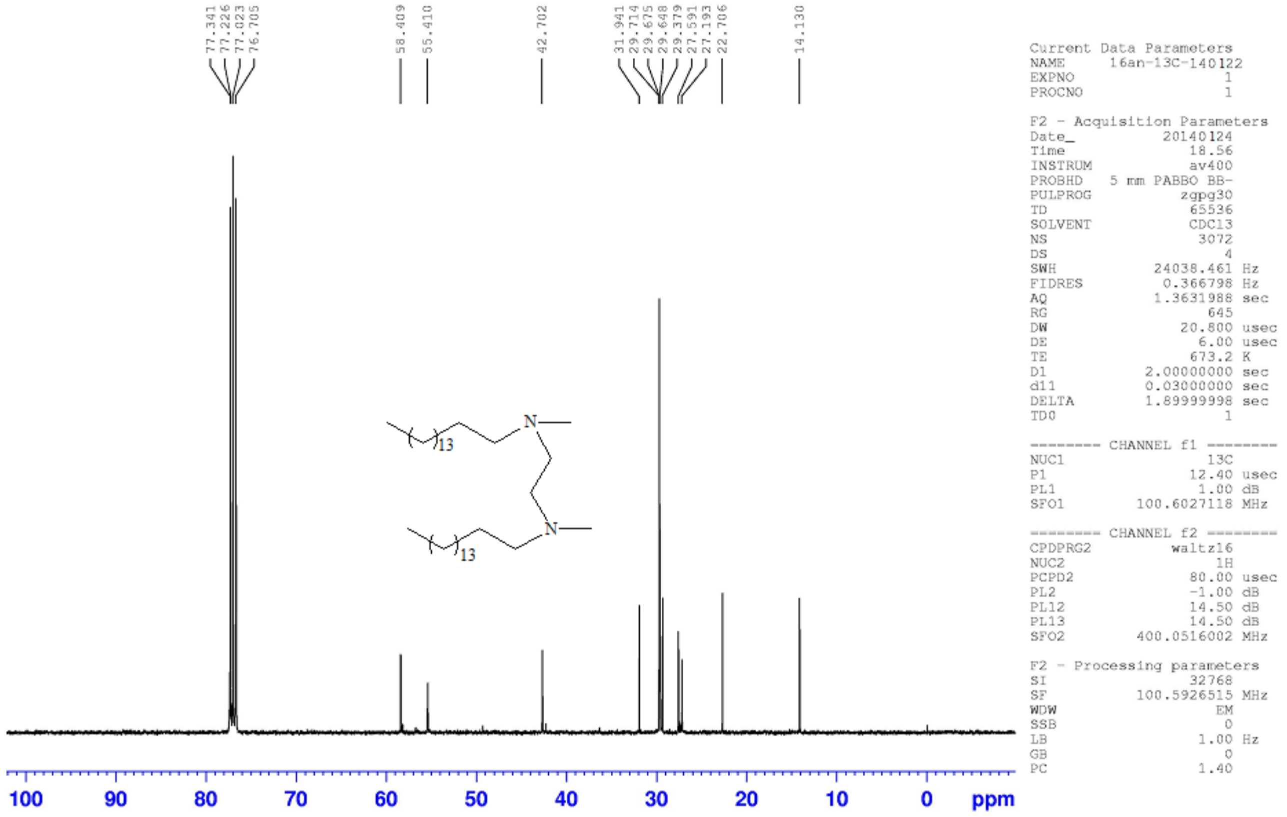

Figure S1. *Cont.*

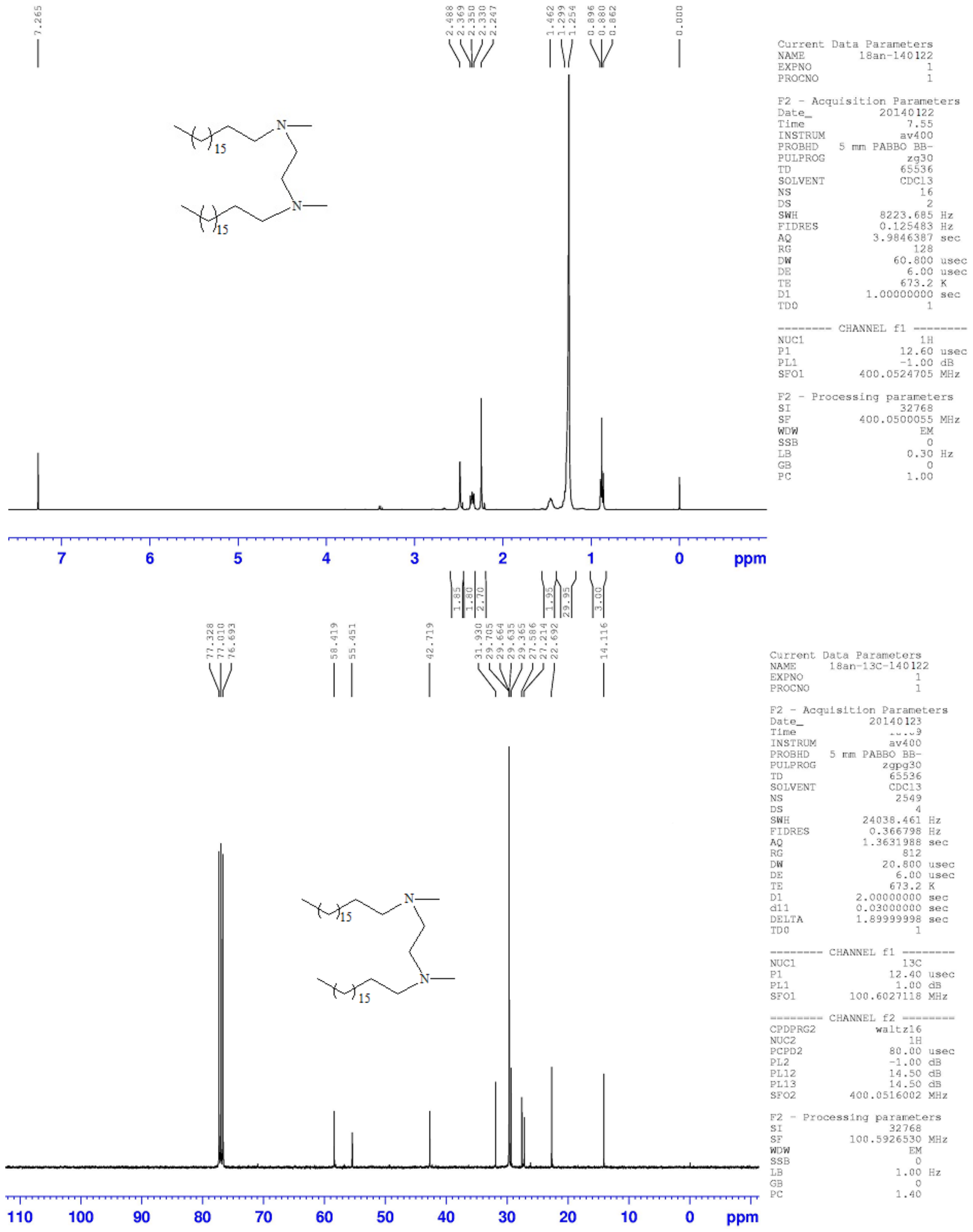

Figure S2. Copies of NMR spectra for 1,2-bis(*N*-methyl-*N*-(3-sulfopropyl)-alkyl ammonium)ethane.

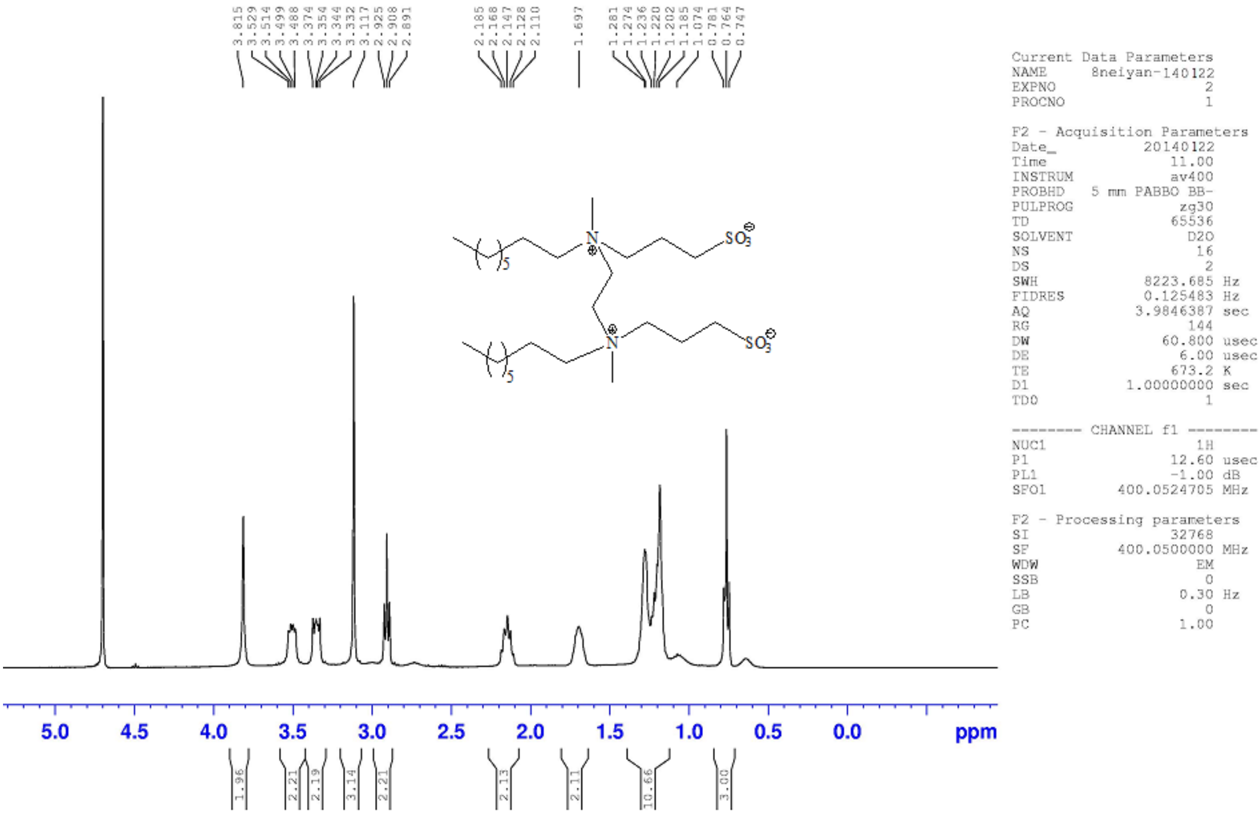

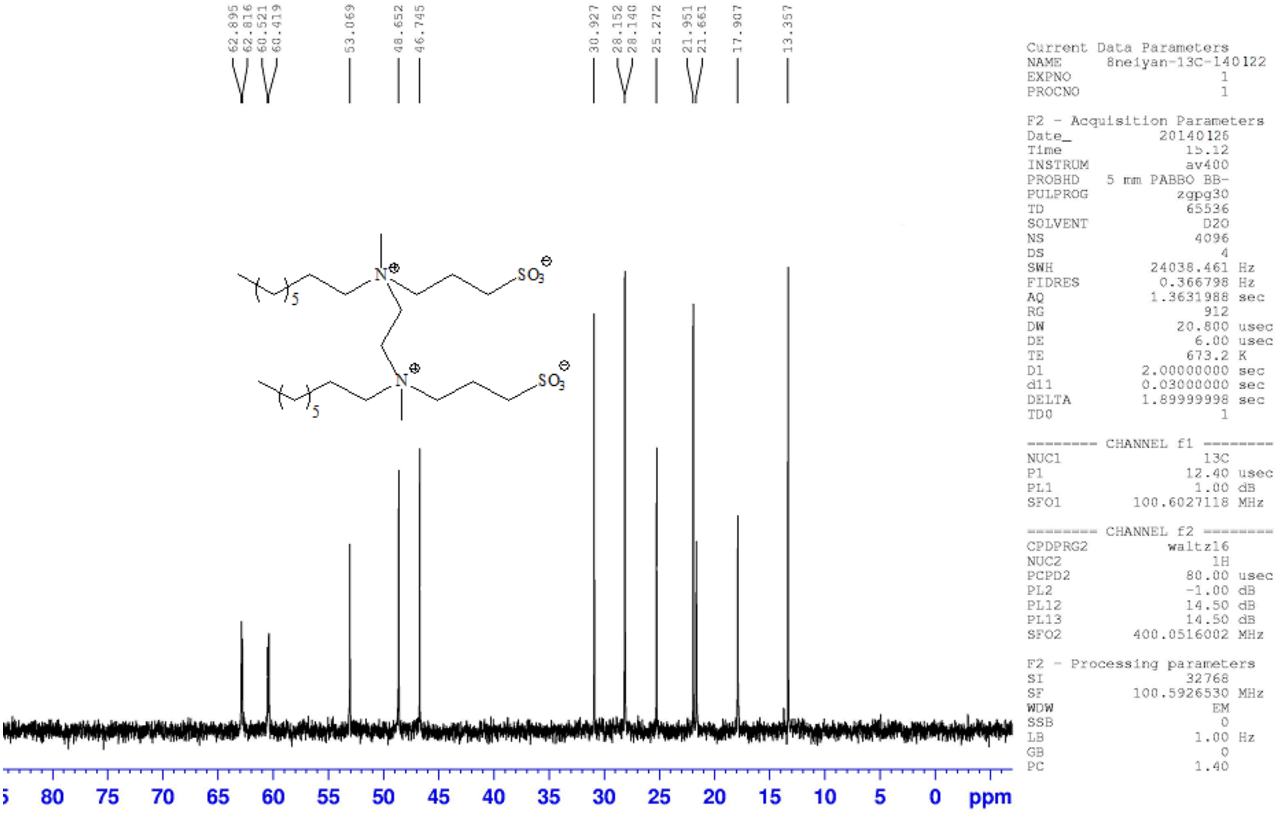

Figure S2. *Cont.*

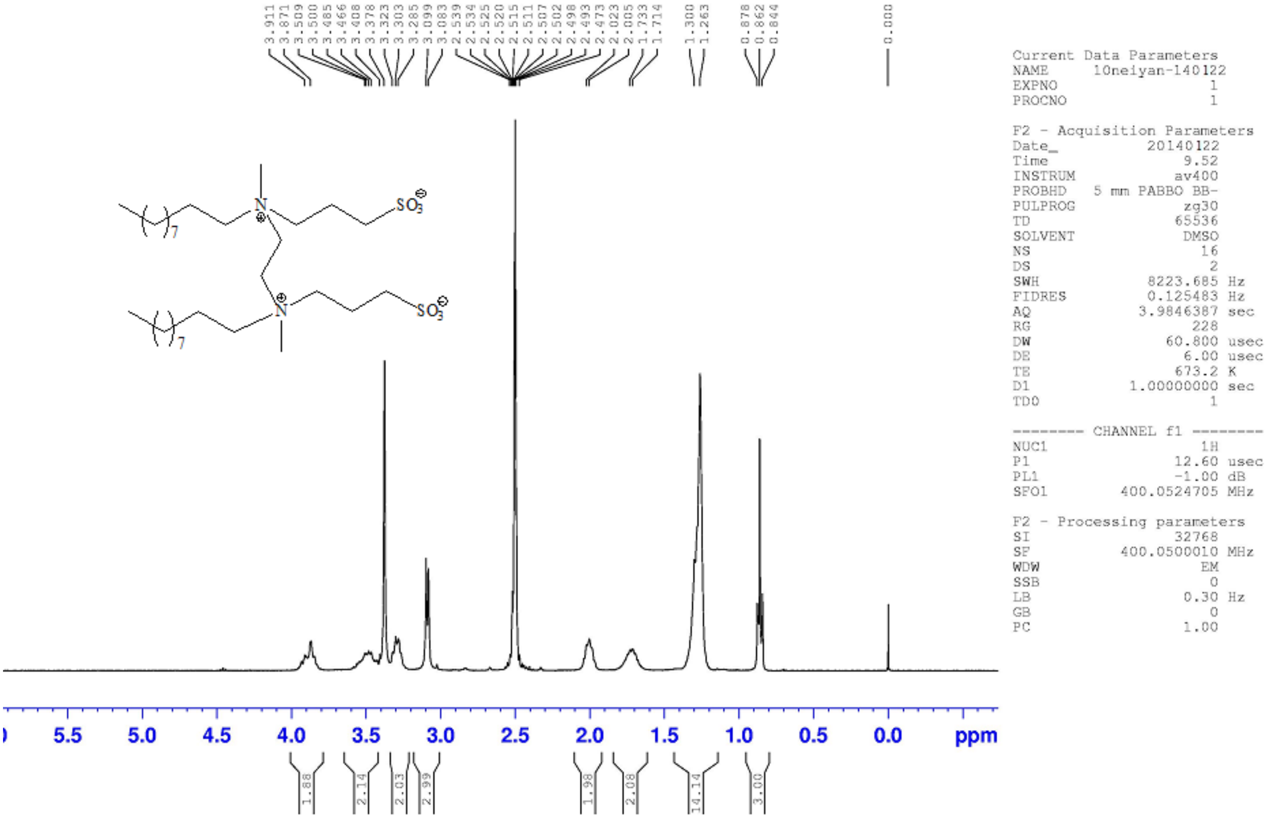

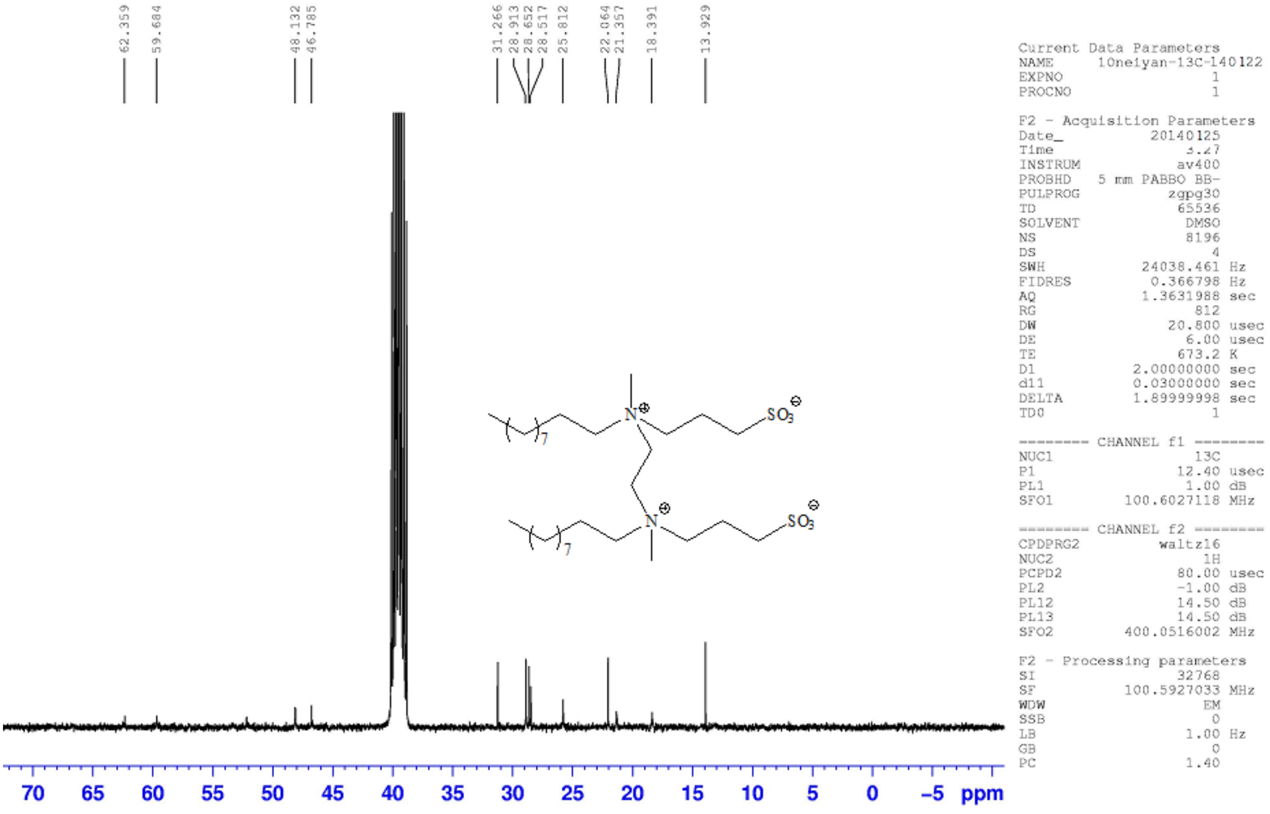

Figure S2. *Cont.*

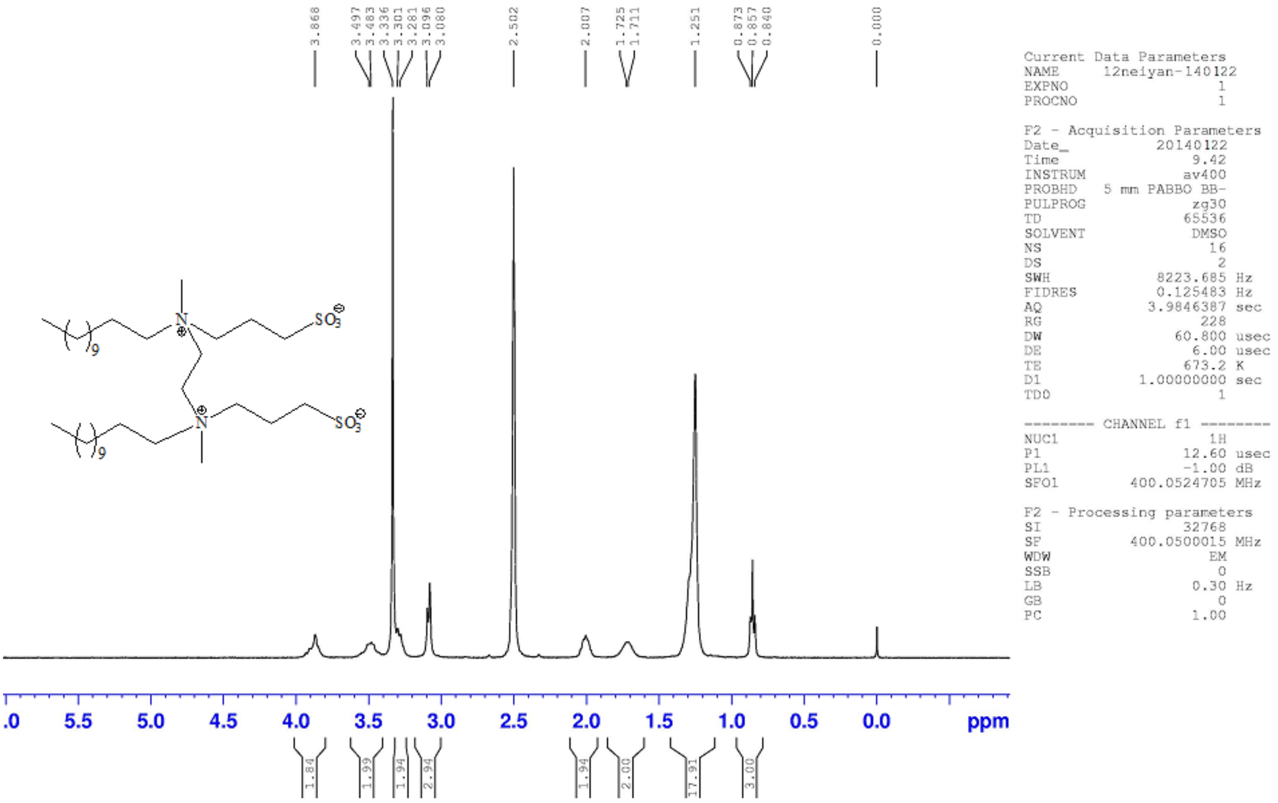

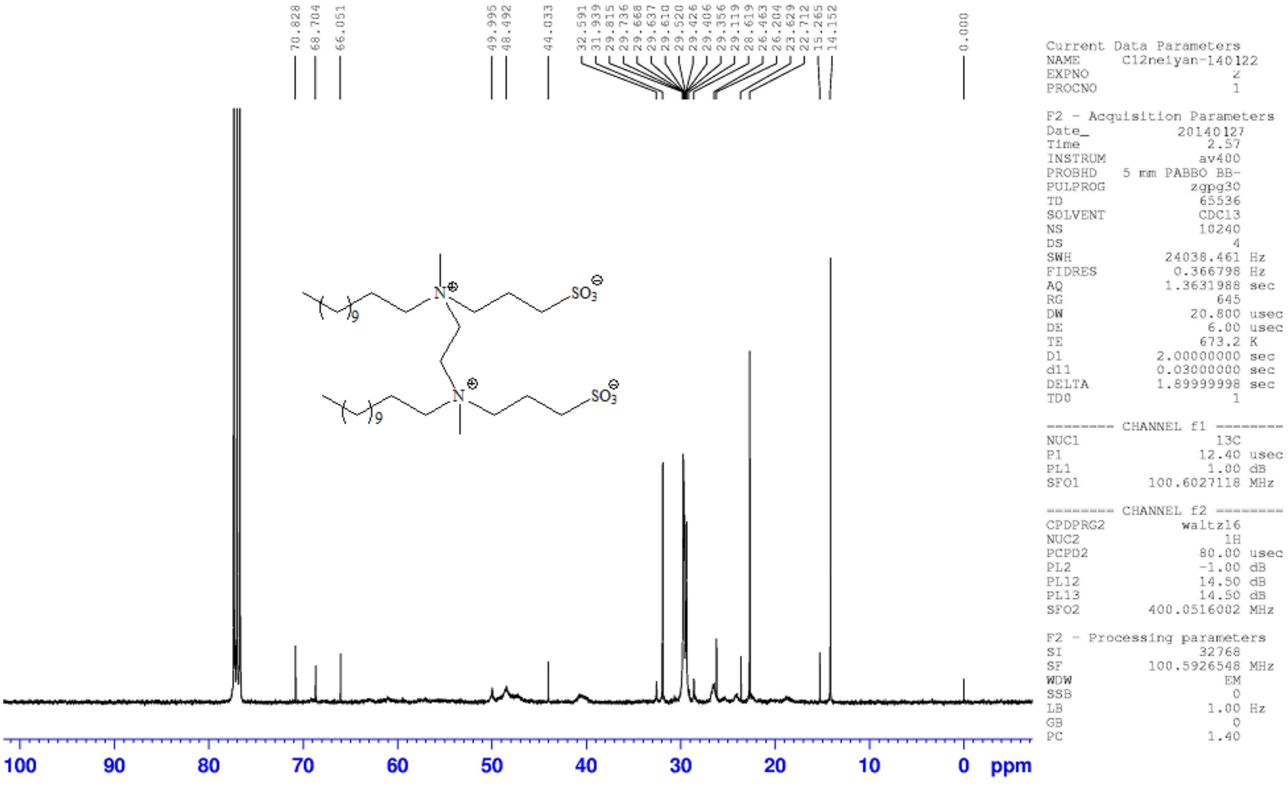

Figure S2. *Cont.*

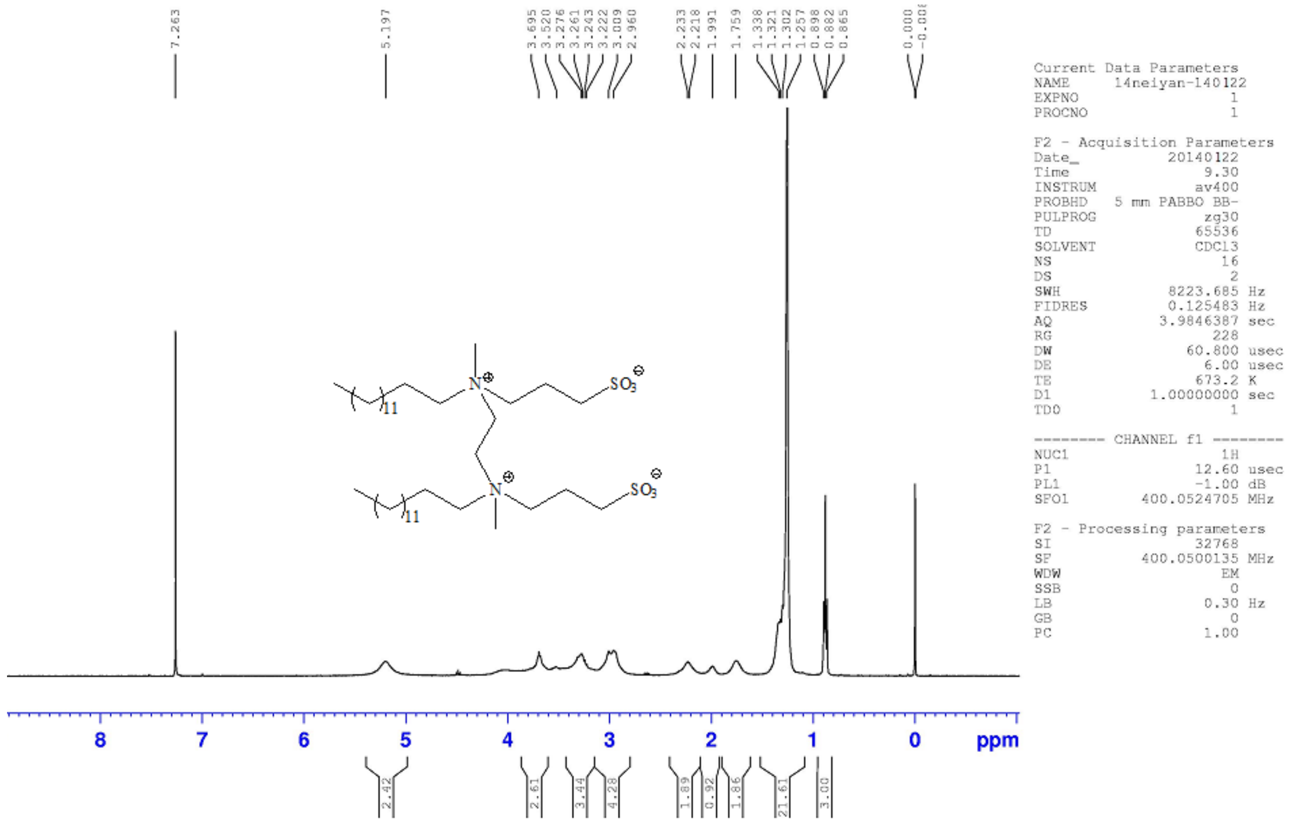

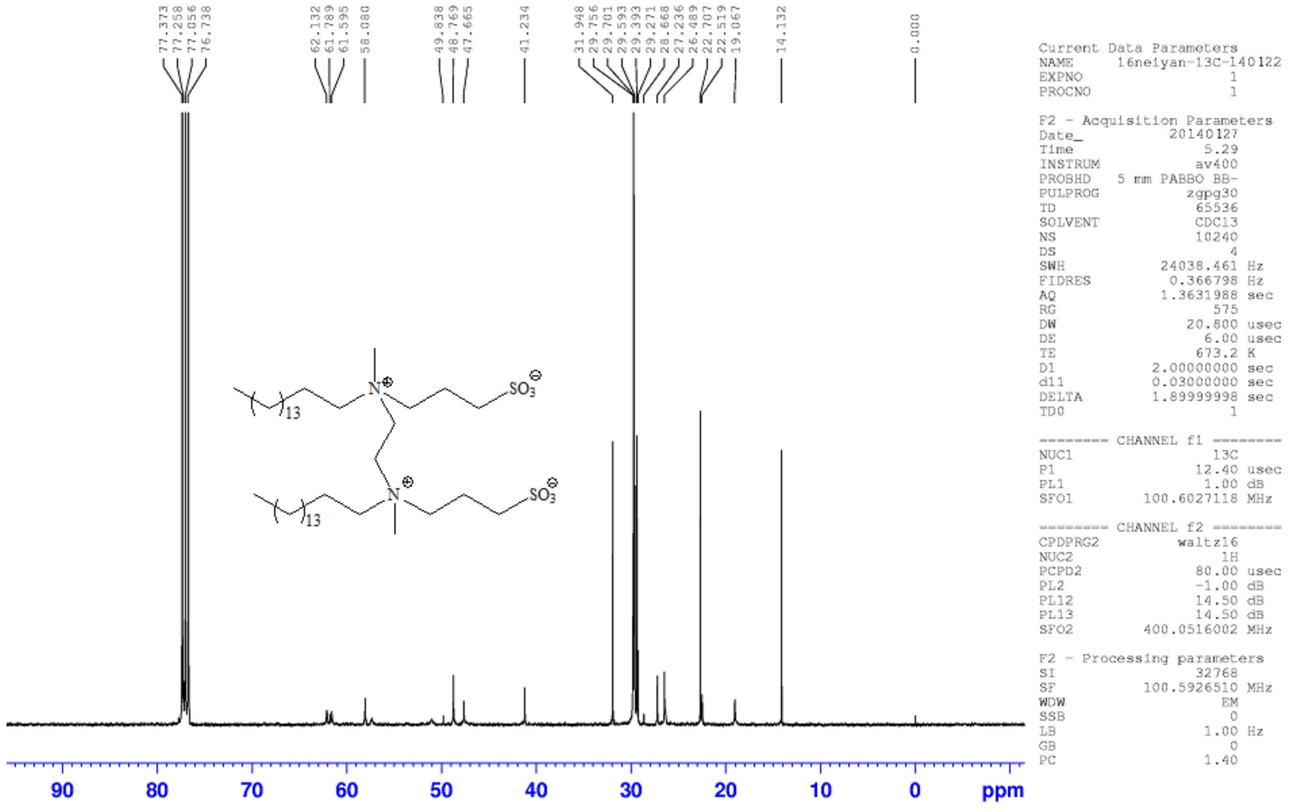

Figure S3. Copies of NMR spectra for GBAILs.

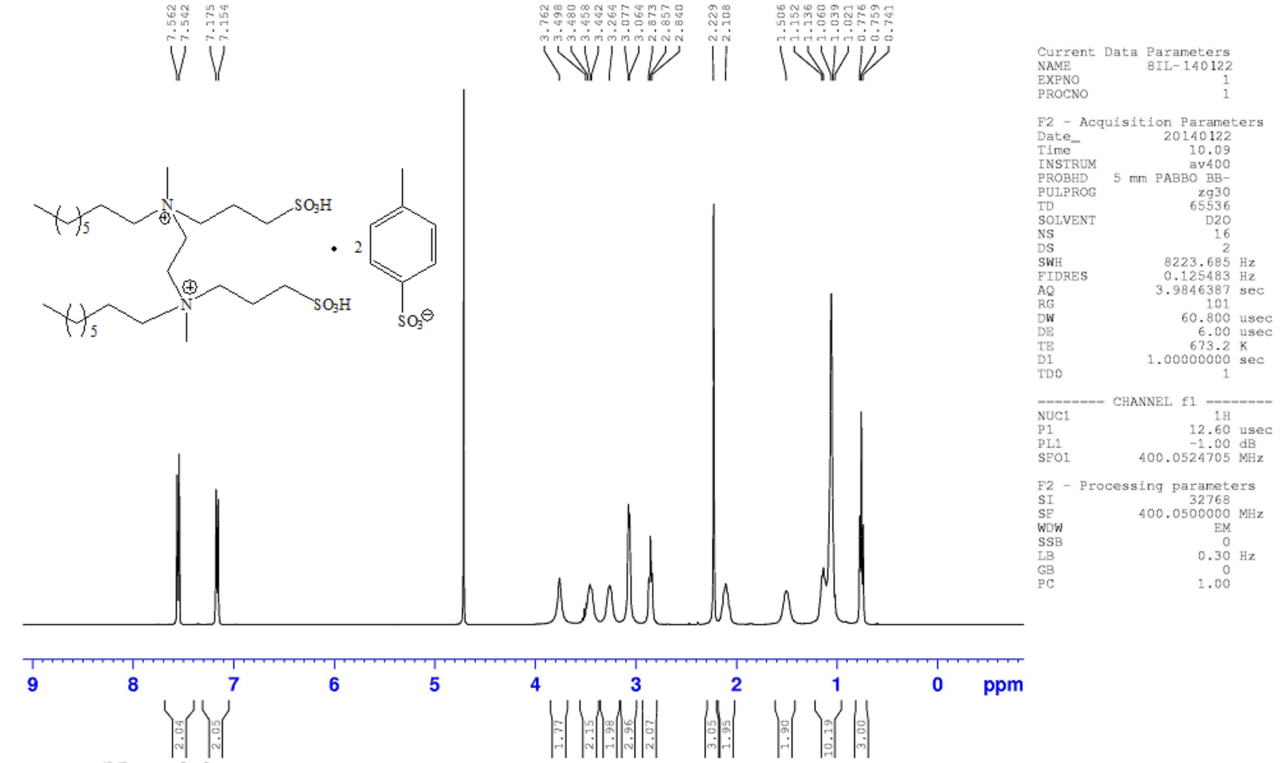

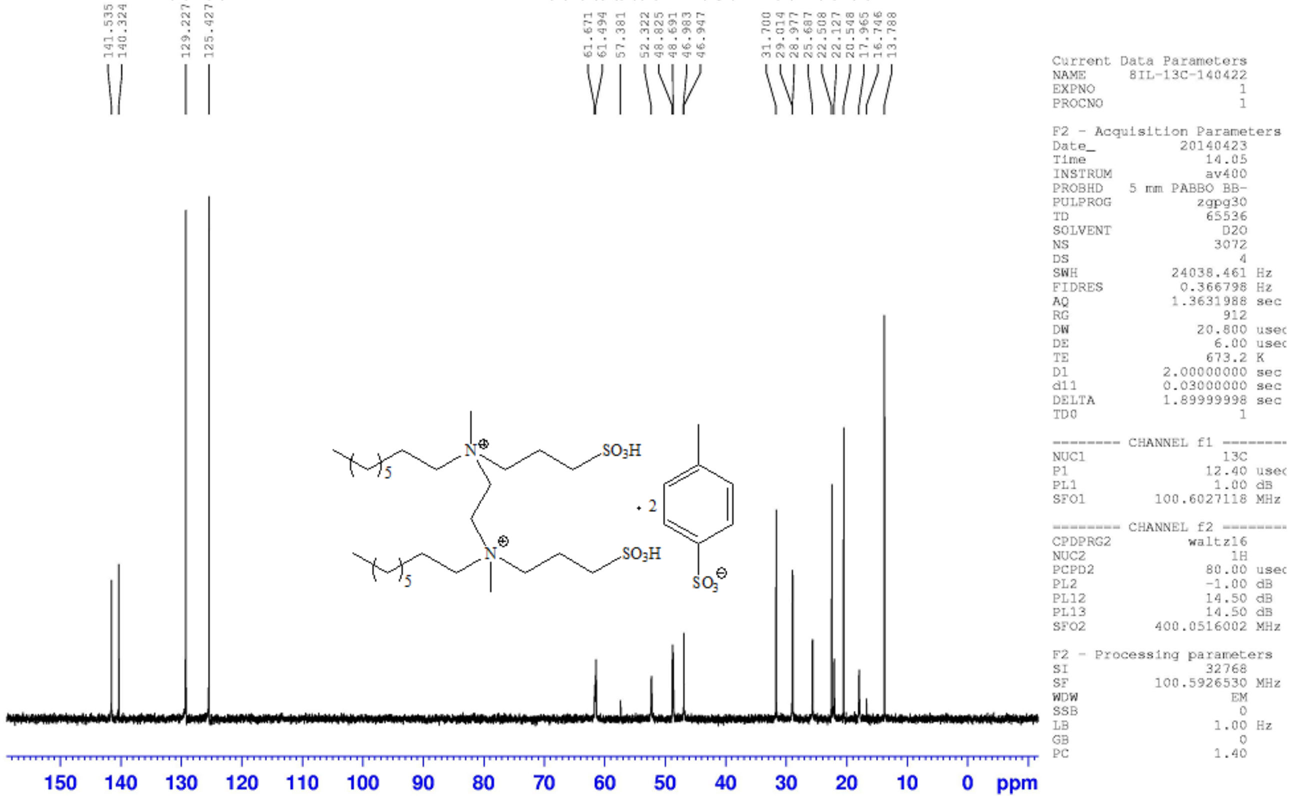

Figure S3. *Cont.*

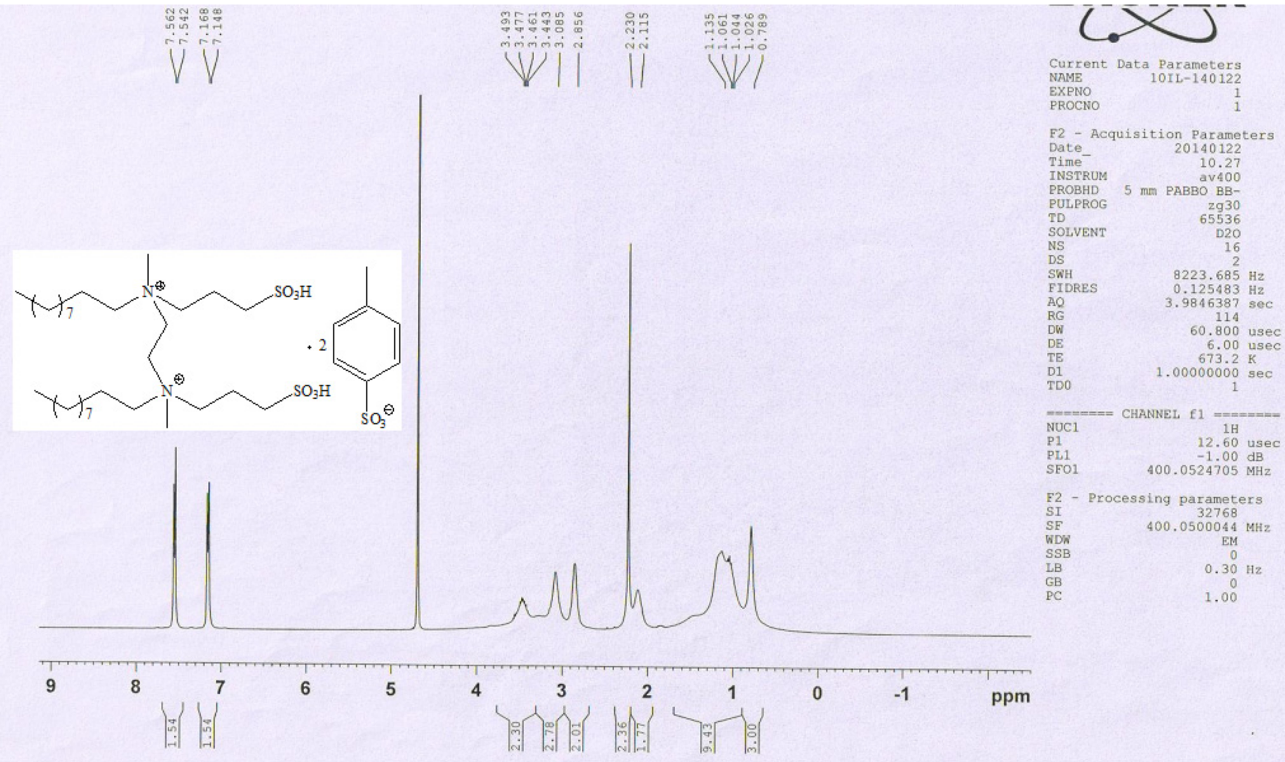

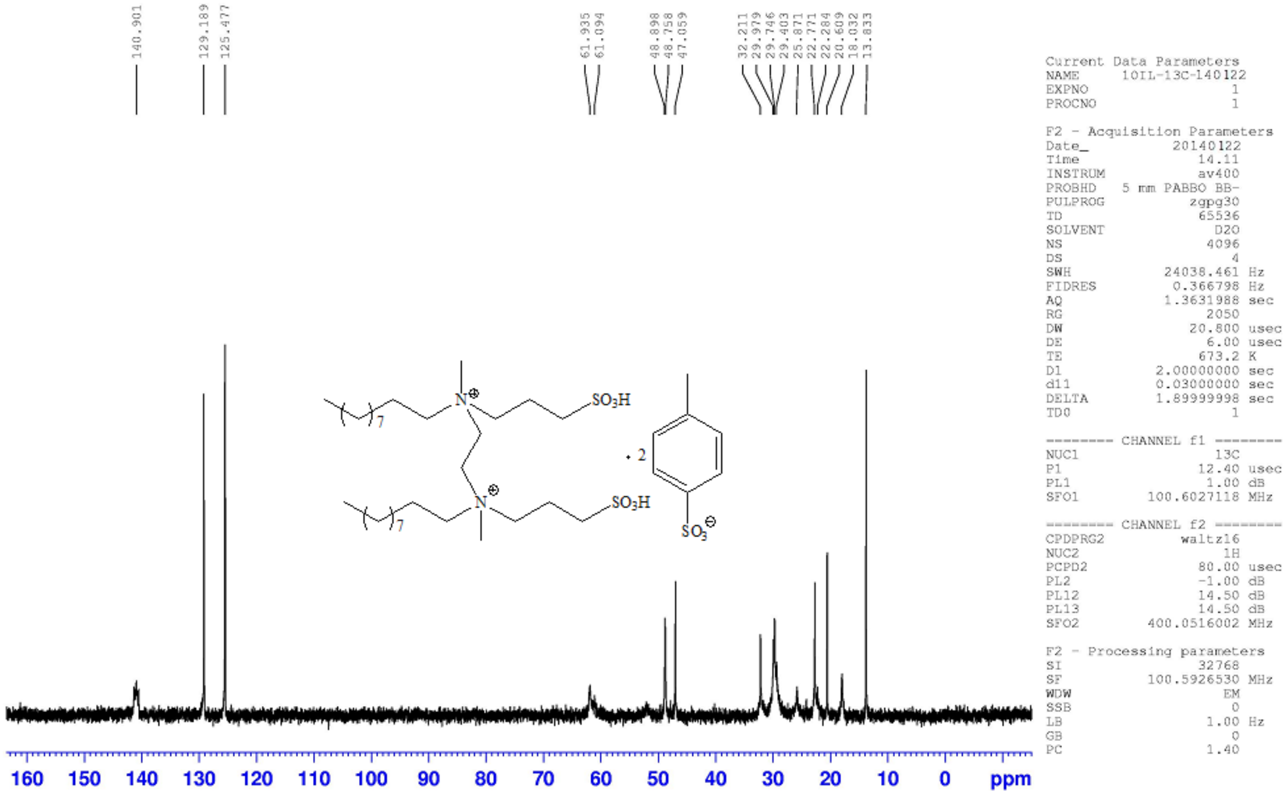

Figure S3. *Cont.*

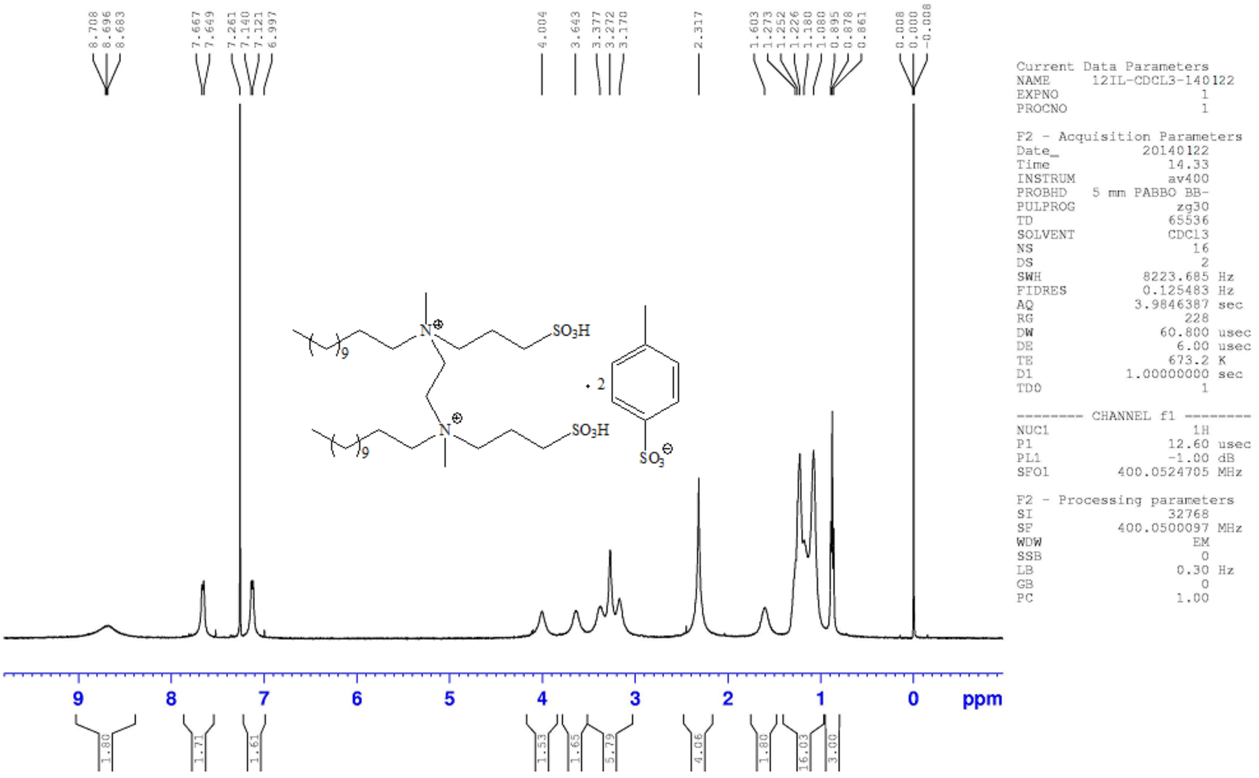

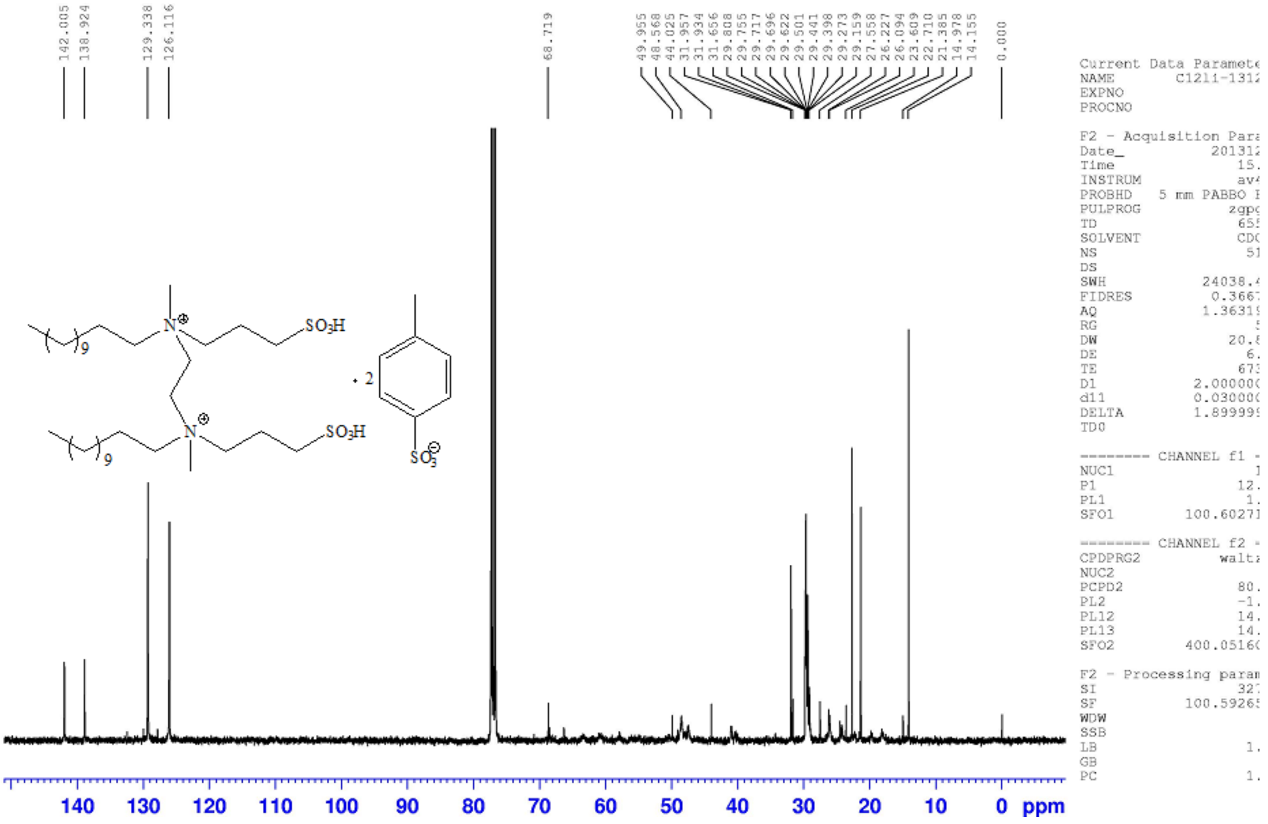

Figure S3. *Cont.*

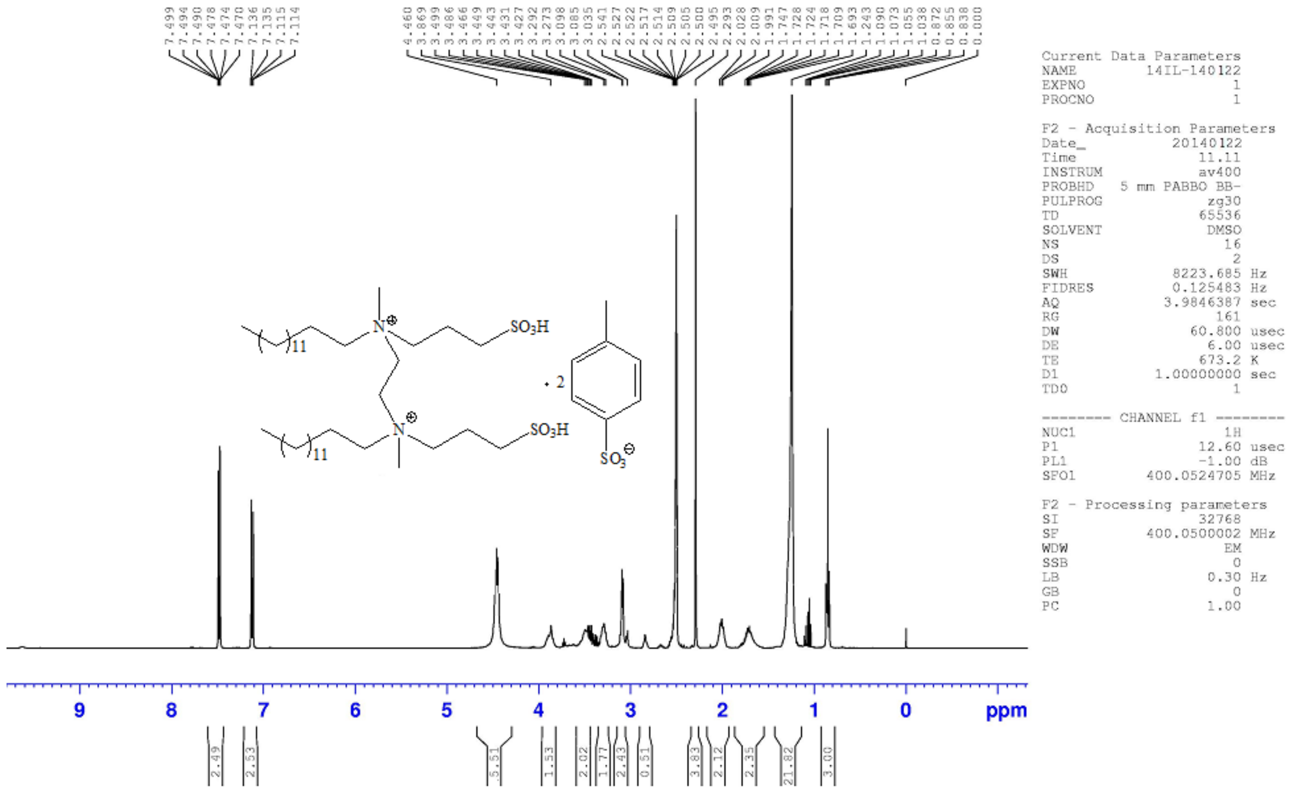

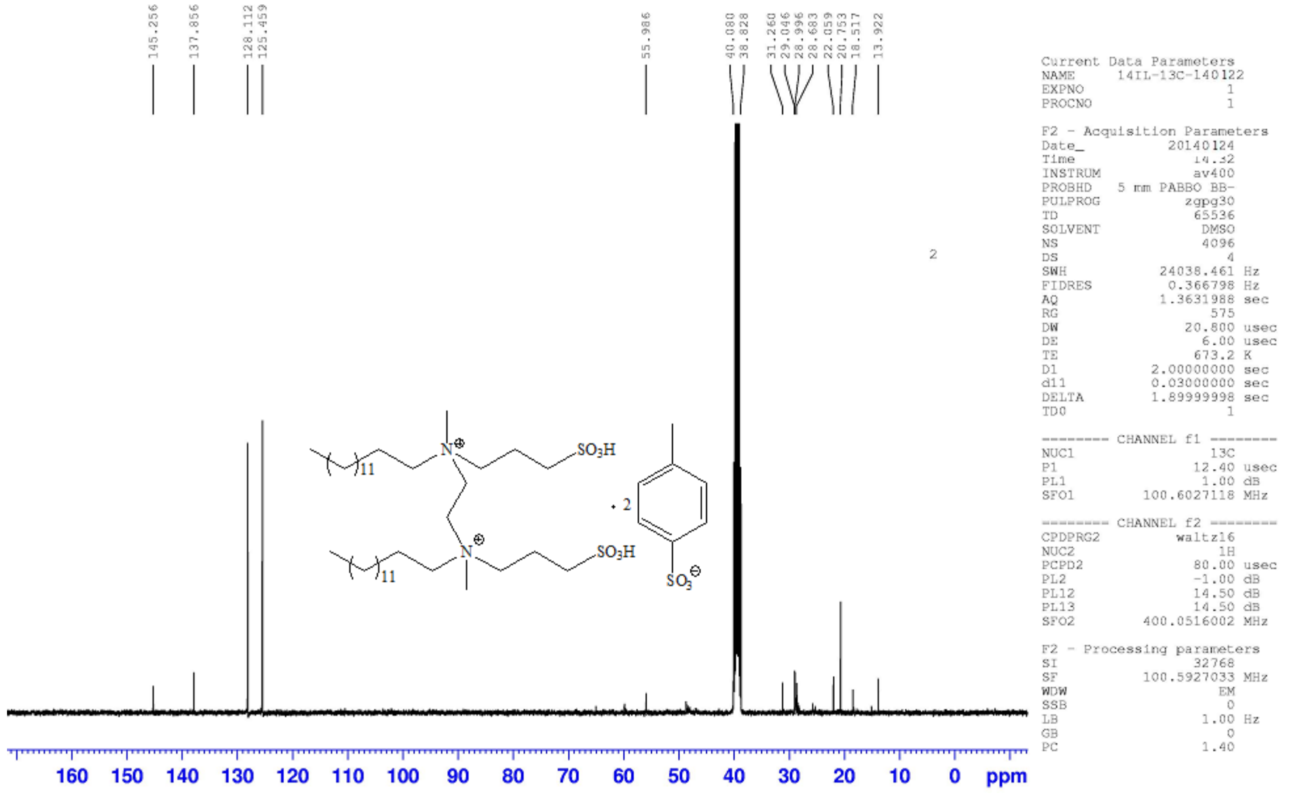

Figure S3. *Cont.*

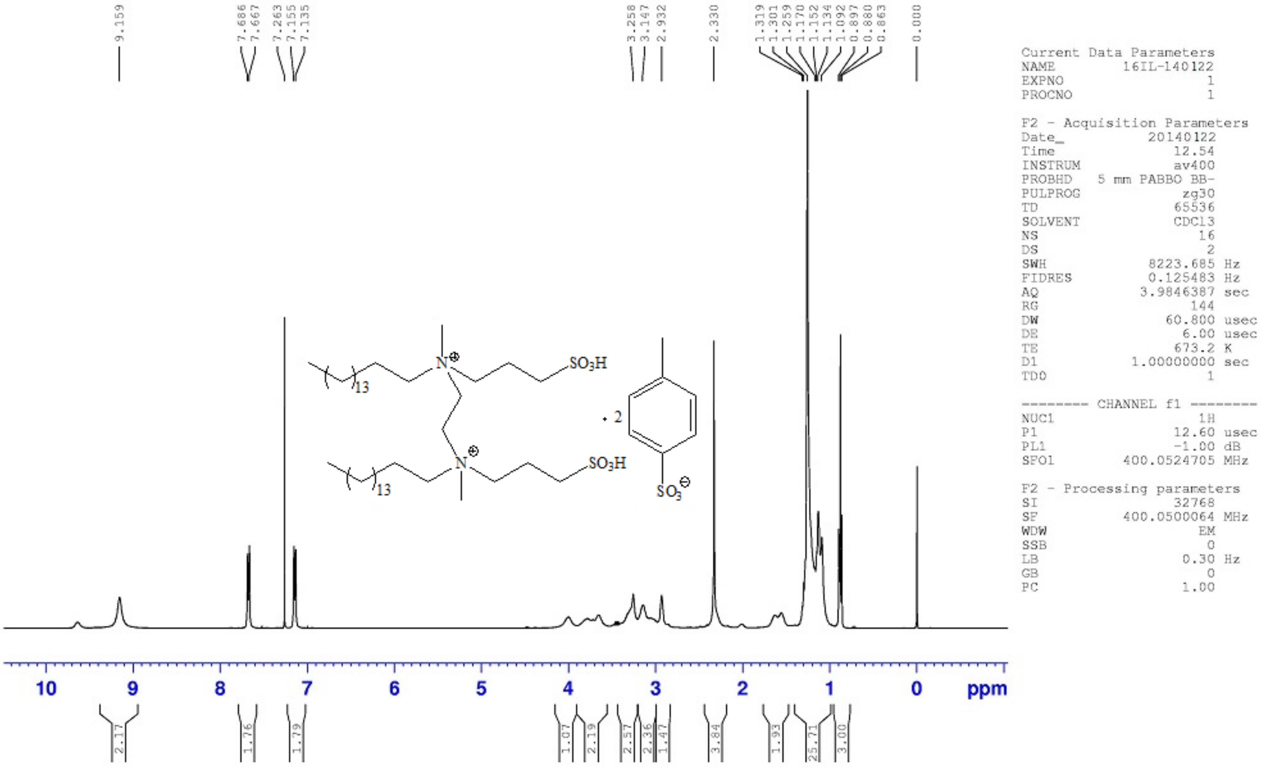

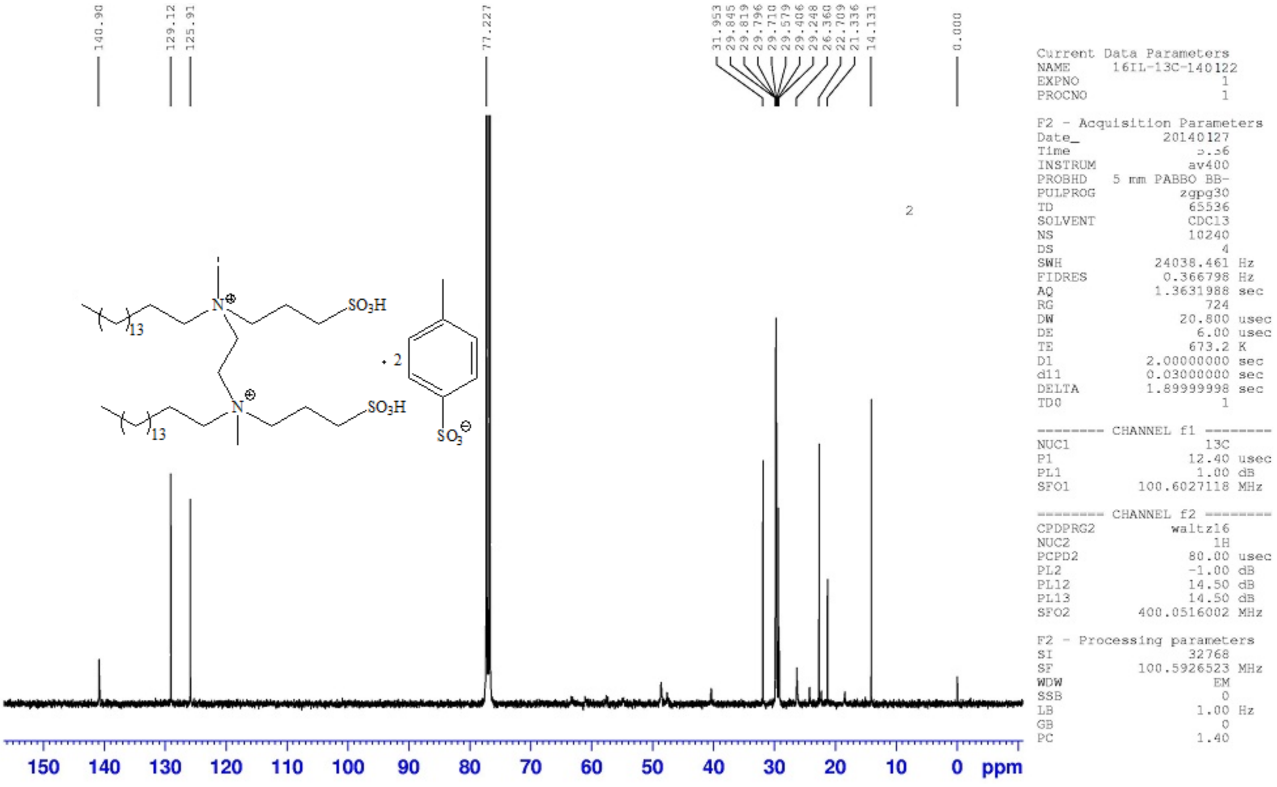

Figure S3. *Cont.*

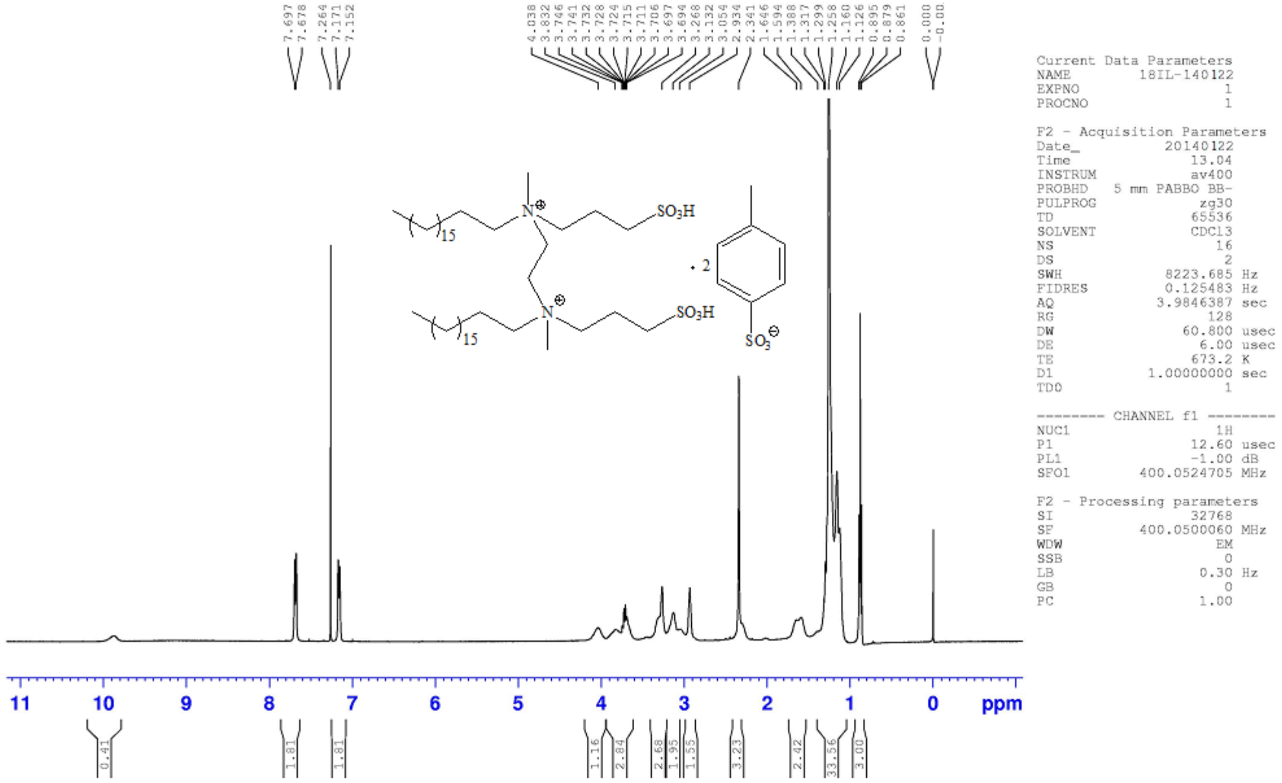

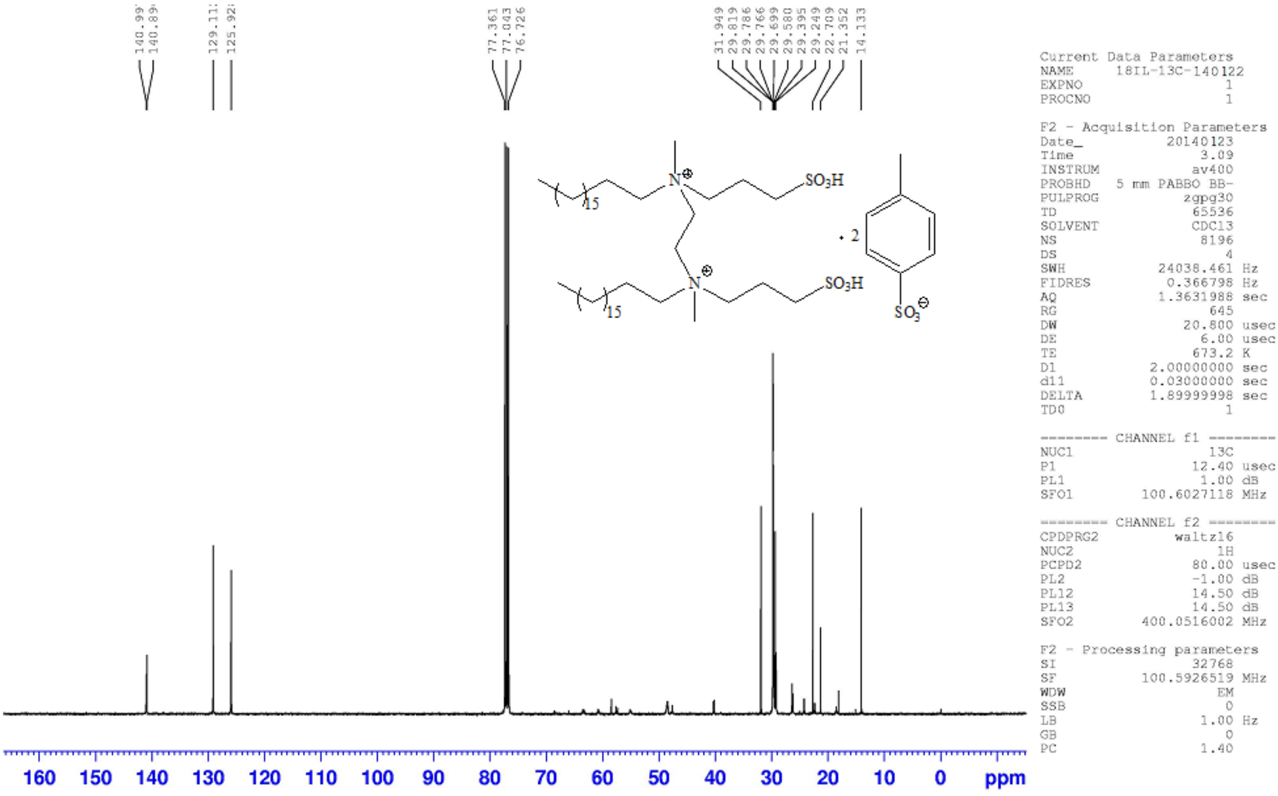

(C) Copies of NMR spectra for the Mannich reaction products

Figure S4. Copies of NMR spectra for the Mannich reaction products.

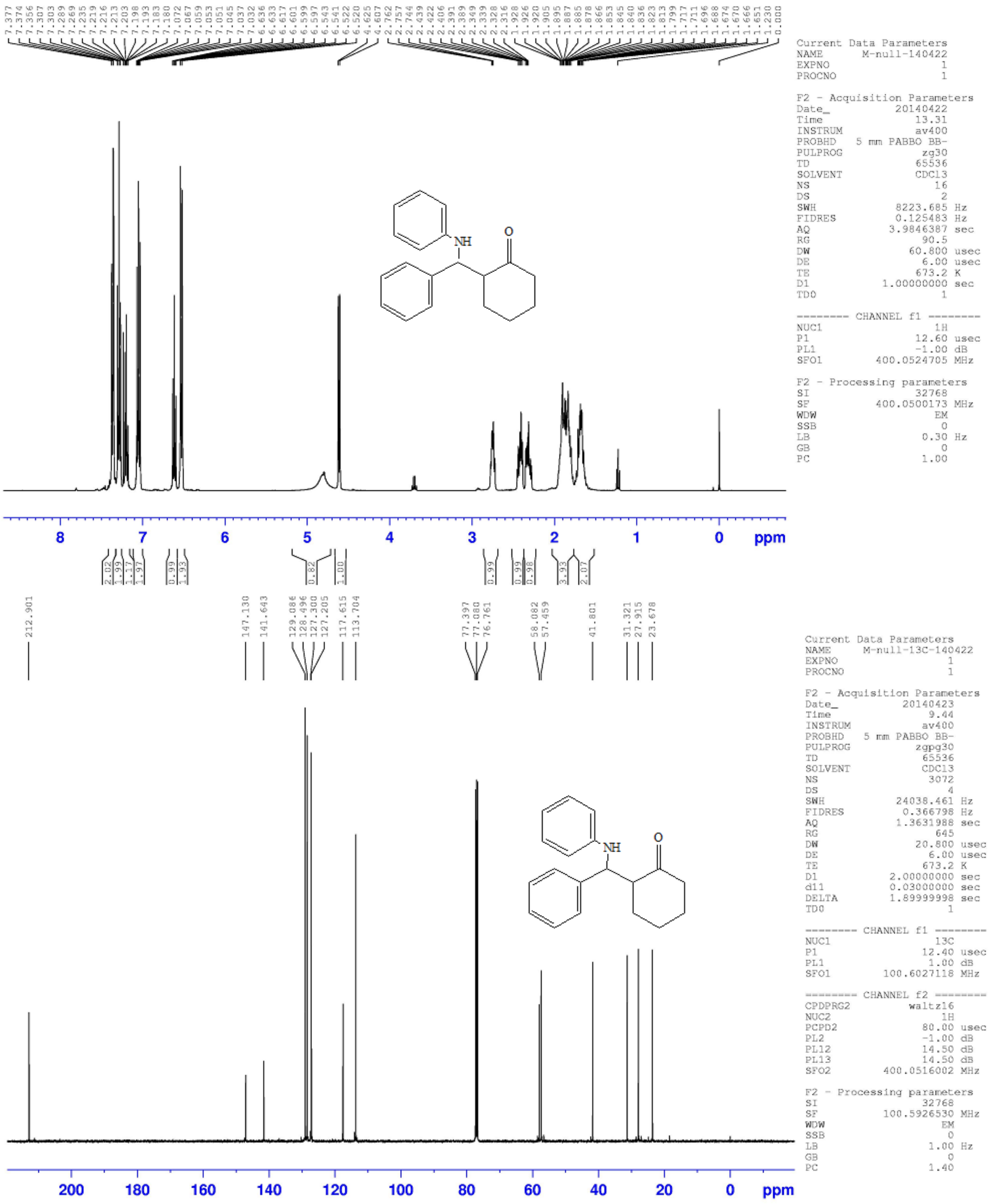

Figure S4. *Cont.*

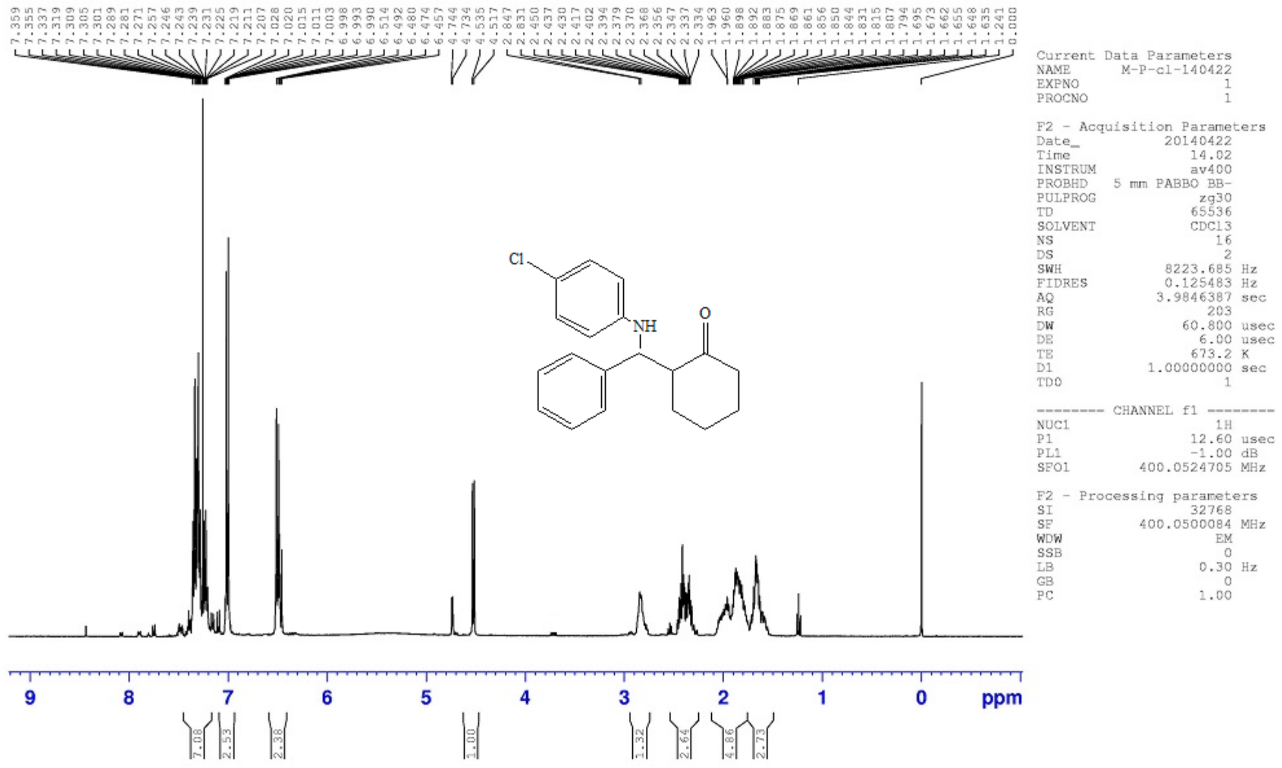

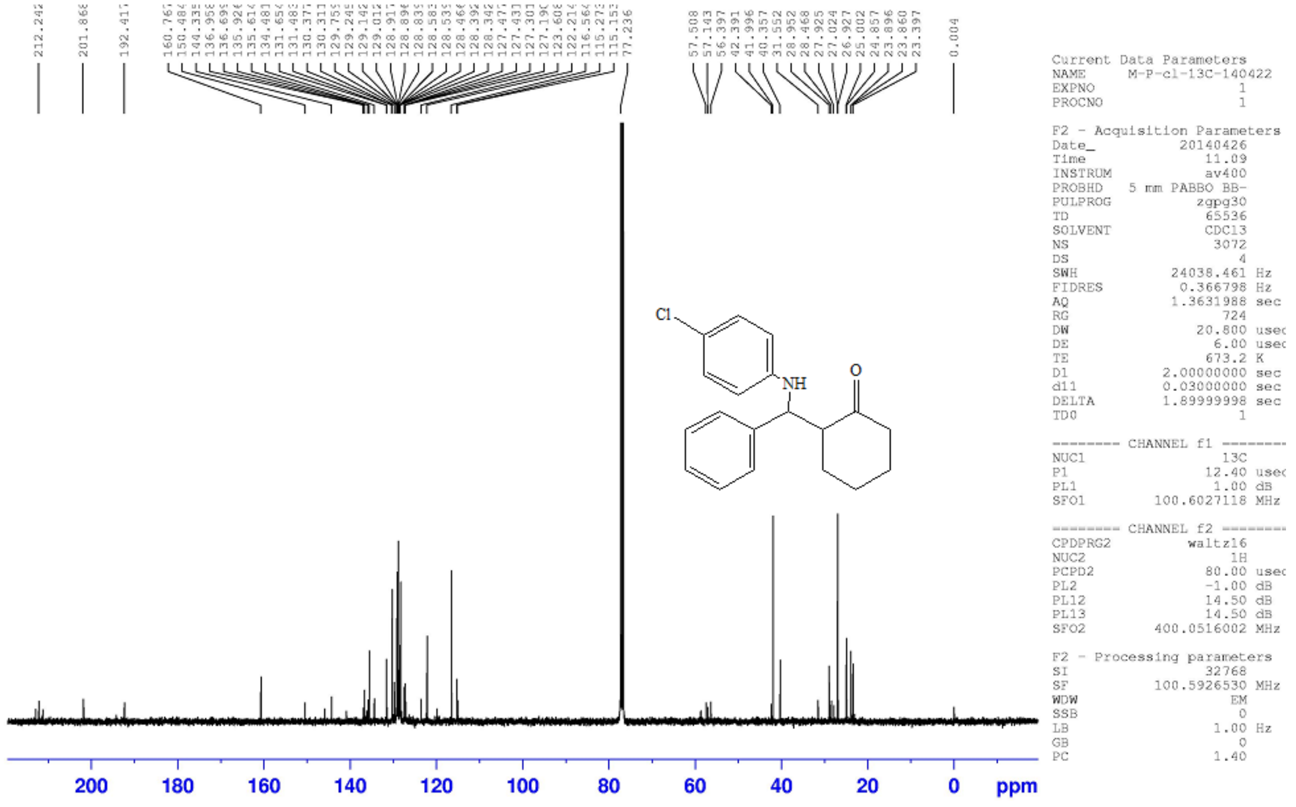

Figure S4. *Cont.*

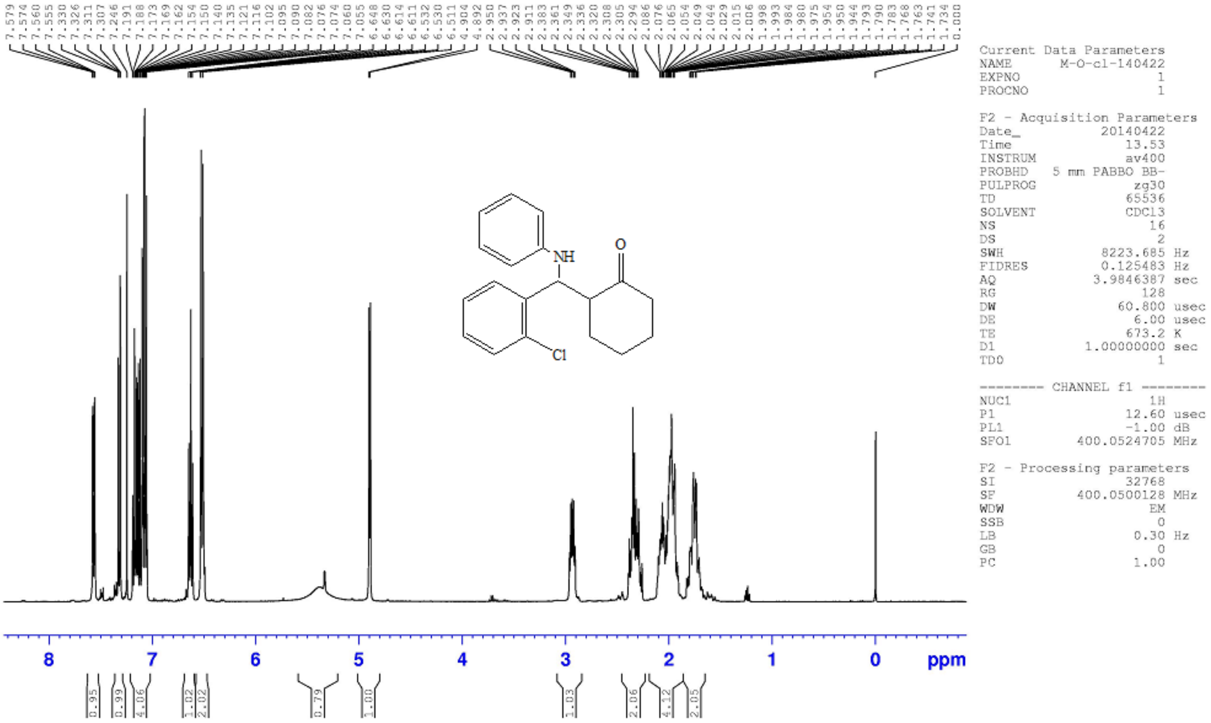

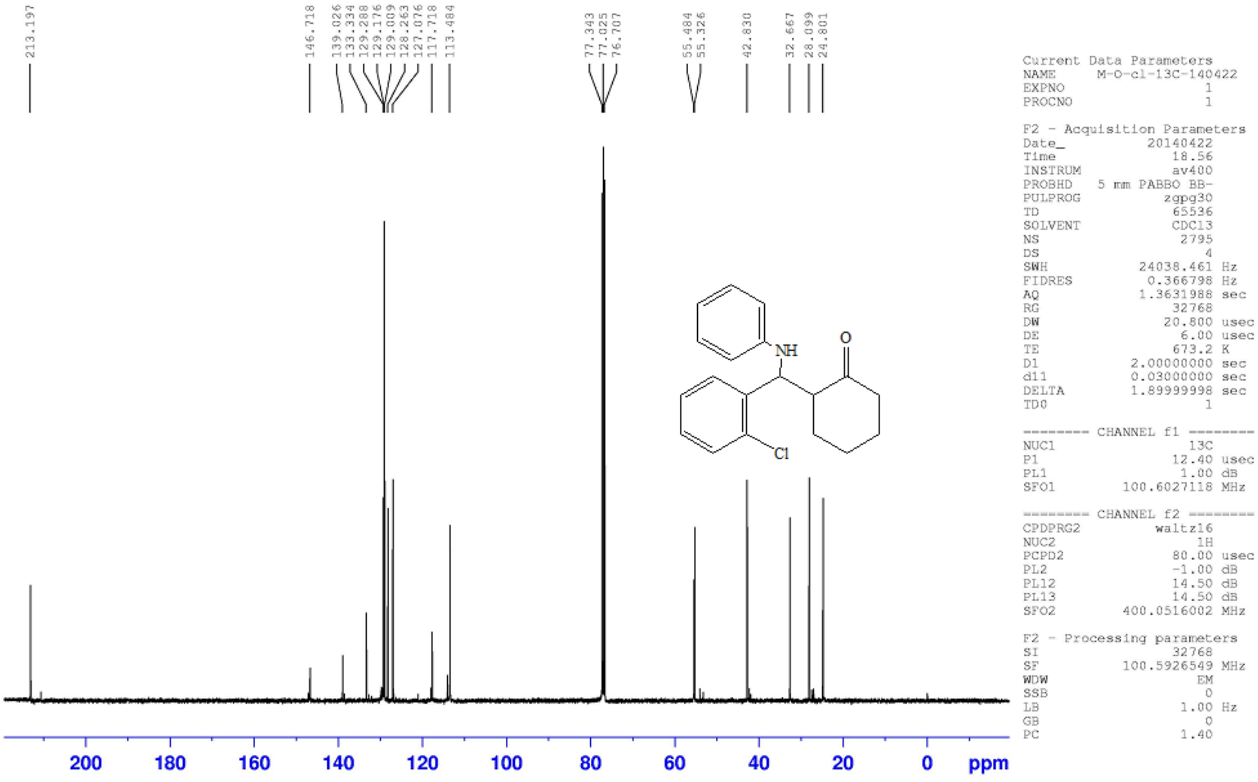

© 2014 by the authors; licensee MDPI, Basel, Switzerland. This article is an open access article distributed under the terms and conditions of the Creative Commons Attribution license (http://creativecommons.org/licenses/by/3.0/).
